# Acquired Drug Resistance Enhances Imidazoquinoline Efflux by P-Glycoprotein

**DOI:** 10.3390/ph14121292

**Published:** 2021-12-10

**Authors:** Anunay J. Pulukuri, Anthony J. Burt, Larissa K. Opp, Colin M. McDowell, Maryam Davaritouchaee, Amy E. Nielsen, Rock J. Mancini

**Affiliations:** 1 Department of Chemistry, Washington State University, Pullman, WA 99164, USA; anunay.pulukuri@wsu.edu (A.J.P.); ajburt@sdsu.edu (A.J.B.); larissa.opp@wsu.edu (L.K.O.); maryam.davaritouchaee@cornell.edu (M.D.); amy.nielsen@wsu.edu (A.E.N.); 2 Department of Chemistry & Biochemistry, San Diego State University, San Diego, CA 92182, USA; 3 School of Molecular Biosciences, Washington State University, Pullman, WA 99164, USA; colin.mcdowell@wsu.edu; 4 Department of Food Science, Cornell University, Ithaca, NY 14853, USA; 5 The Gene & Linda Voiland School of Chemical Engineering and Bioengineering, Washington State University, Pullman, WA 99164, USA

**Keywords:** Imiquimod, drug efflux, multidrug resistance, immunotherapy, Toll-Like Receptor

## Abstract

Multidrug-Resistant (MDR) cancers attenuate chemotherapeutic efficacy through drug efflux, a process that transports drugs from within a cell to the extracellular space via ABC (ATP-Binding Cassette) transporters, including P-glycoprotein 1 (P-gp or ABCB1/MDR1). Conversely, Toll-Like Receptor (TLR) agonist immunotherapies modulate activity of tumor-infiltrating immune cells in local proximity to cancer cells and could, therefore, benefit from the enhanced drug efflux in MDR cancers. However, the effect of acquired drug resistance on TLR agonist efflux is largely unknown. We begin to address this by investigating P-gp mediated efflux of TLR 7/8 agonists. First, we used functionalized liposomes to determine that imidazoquinoline TLR agonists Imiquimod, Resiquimod, and Gardiquimod are substrates for P-gp. Interestingly, the least potent imidazoquinoline (Imiquimod) was the best P-gp substrate. Next, we compared imidazoquinoline efflux in MDR cancer cell lines with enhanced P-gp expression relative to parent cancer cell lines. Using P-gp competitive substrates and inhibitors, we observed that imidazoquinoline efflux occurs through P-gp and, for Imiquimod, is enhanced as a consequence of acquired drug resistance. This suggests that enhancing efflux susceptibility could be an important consideration in the rational design of next generation immunotherapies that modulate activity of tumor-infiltrating immune cells.

## 1. Introduction

Multidrug-Resistant (MDR) cancers are implicated in over 90% of metastatic cancer deaths, including melanomas, breast cancer, and prostate cancer [1]. A general trend among MDR cancers is enhanced drug efflux, a term describing the expression of transport proteins that traffic drugs from within a cell to the extracellular space, thereby lowering intracellular drug concentration [2]. In MDR cancers, drug efflux is facilitated by the ABC (ATP-Binding cassette) superfamily which consists of at least 48 distinct transport proteins [3,4]. Of these, P-glycoprotein (P-gp or ABCB1/MDR1) was the first discovered [5,6] and is the most well-studied [7,8]. P-gp is particularly promiscuous, transporting structurally diverse compounds with minimal correlation other than a weak association with hydrophobicity [9,10,11,12,13]. Entire classes of chemotherapeutics, such as taxanes or anthracyclines, are substrates for P-gp-mediated drug efflux [14,15], which makes defeating this mechanism of chemoresistance particularly challenging [16]. However, this same promiscuity could be useful for enhancing extracellular concentrations of small molecule immunomodulator drugs that target bystander cells in the tumor microenvironment, such as tumor-infiltrating lymphocyte (TIL) and tumor-associated macrophage (TAM) populations. As such, we were interested in examining the effect of MDR on the ability of small molecule immunomodulators to undergo drug efflux.

From the first empirical whole-organism Toll-Like Receptor (TLR) agonists in the late 1800s [17,18], a range of proinflammatory immunotherapeutics have emerged with mechanisms of action fundamentally orthogonal to P-gp-mediated drug resistance. Within this drug class, imidazoquinoline TLR 7/8 agonists promote TIL and TAM reprogramming along tumor-suppressing axes [19], resulting in a myriad of immune-mediated anti-cancer effects [20,21,22,23,24,25] in both mono [26] and combination [27] therapies. To achieve these effects, while also limiting systemic inflammatory toxicity, imidazoquinolines are typically administered locally, via topical application or intratumoral injection. This has led our group [28,29,30] and others [31,32,33,34] to develop prodrug delivery strategies that liberate imidazoquinolines, either within the tumor microenvironment, or within cancer cells themselves, by intracellular immunostimulant accumulation followed by drug efflux. Although these strategies have the potential to facilitate delivery of imidazoquinolines to TILs and TAMs, the results also point to drug efflux as a potential rate-limiting step in immune cell activation for imidazoquinolines liberated within cancer cells. This suggests that MDR cancer cells with enhanced transport protein expression could be more susceptible to small-molecule immunotherapeutics. However, the effect of acquired MDR on imidazoquinoline immunotherapeutic efflux has never been explored, apart from a recent study that exclusively examines Resiquimod trafficking [35]. As such, we hypothesized that identifying a pathway for imidazoquinoline efflux and establishing the effects of acquired MDR on efflux would inform the design of the next generation of small-molecule immunotherapeutic prodrugs.

**Figure 1:**
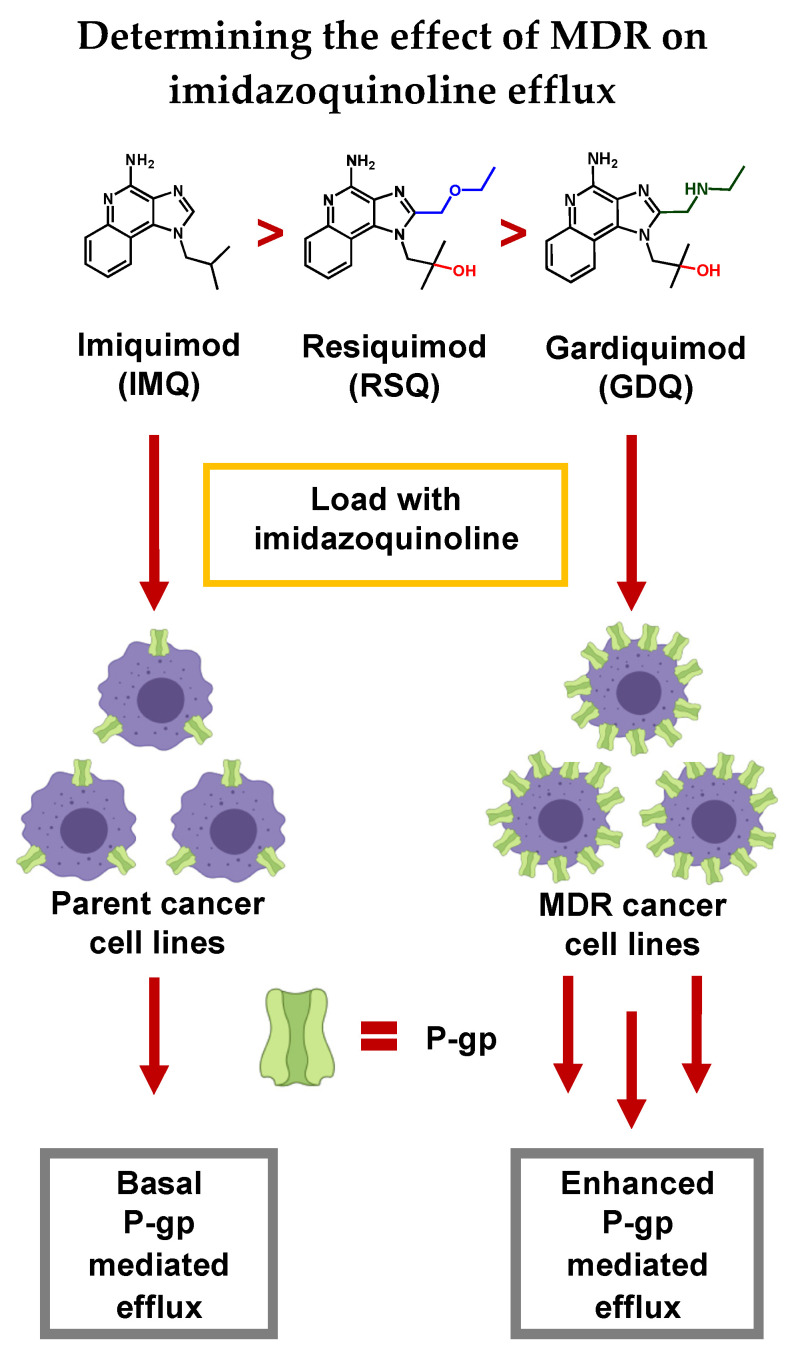
In this study we establish that Imiquimod (IMQ), Resiquimod (RSQ), and Gardiquimod (GDQ) are substrates of P-gp, and compare P-gp-mediated efflux between Multidrug-Resistant (MDR) cancer cell lines relative to parent cell lines. We also conclude that some efflux likely occurs through other transport proteins as well as passive transport into the extracellular space.

Herein, we demonstrate that imidazoquinolines are highly variable as substrates for P-gp efflux, both in a membrane vesicle model, as well as in vitro across a range of MDR cancer cells (Figure 1). Overall, we find that efflux is significantly enhanced by the MDR phenotype depending on both the type of cancer and the substituent variation regarding N1 and C2 locations on the imidazoquinoline structure itself.

## 2. Results

### 2.1. Imiquimod, Resiquimod, and Gardiquimod Are Substrates of P-gp

**Figure 2:**
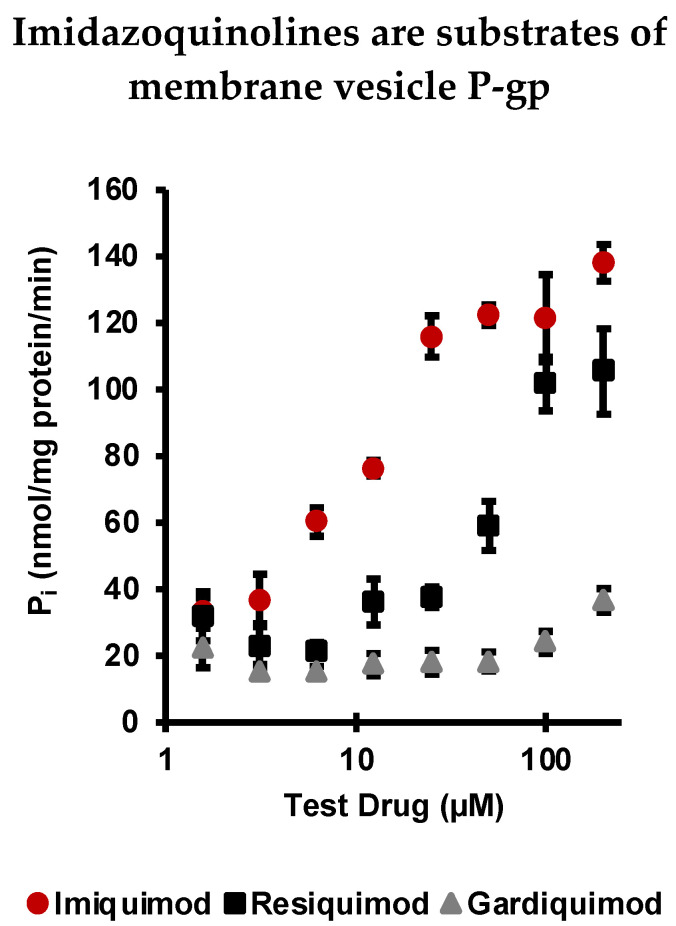
ATPase assay with IMQ, RSQ, and GDQ. In the activation test, IMQ, RSQ, and GDQ all stimulated vanadate-sensitive ATPase activity above baseline, confirming that the imidazoquinolines are substrates of P-gp. Error bars are standard deviation from the mean of experiments repeated in duplicate.

To determine if imidazoquinolines are substrates of P-gp, an ATPase assay using purified membrane vesicles expressing P-gp was performed (Figure 2). All three imidazoquinolines were tested at the same concentrations, from 1.56 to 200 µM. Concentration dependent activation of P-gp ATP (quantified as liberated P_i_) was detectable down to 6.25 µM for IMQ. RSQ also stimulated P-gp at higher concentrations, whereas GDQ was a poor substrate by comparison, only liberating P_i_ above baseline at the highest concentrations tested. That said, each imidazoquinoline did liberate P_i_ in the activation test, confirming that all are P-gp substrates, albeit to varying degrees. Based upon this result, we calculated imidazoquinoline P-gp substrate affinity as (Table 1): IMQ (K_D_ = 7.66 μM) > RSQ (K_D_ = 24.37 μM) > GDQ (N.D.). These results were further confirmed using SwissADME, which predicted each imidazoquinoline to be a substrate for P-gp [36]. None of the three imidazoquinolines significantly inhibited maximal vanadate-sensitive ATPase activity when tested as inhibitors, an important point because P-gp-mediated drug efflux occurs through multiple mechanisms (Figure S3) [37].

### 2.2. MDR Cancer Cell Lines Enhance P-gp Expression

**Figure 3:**
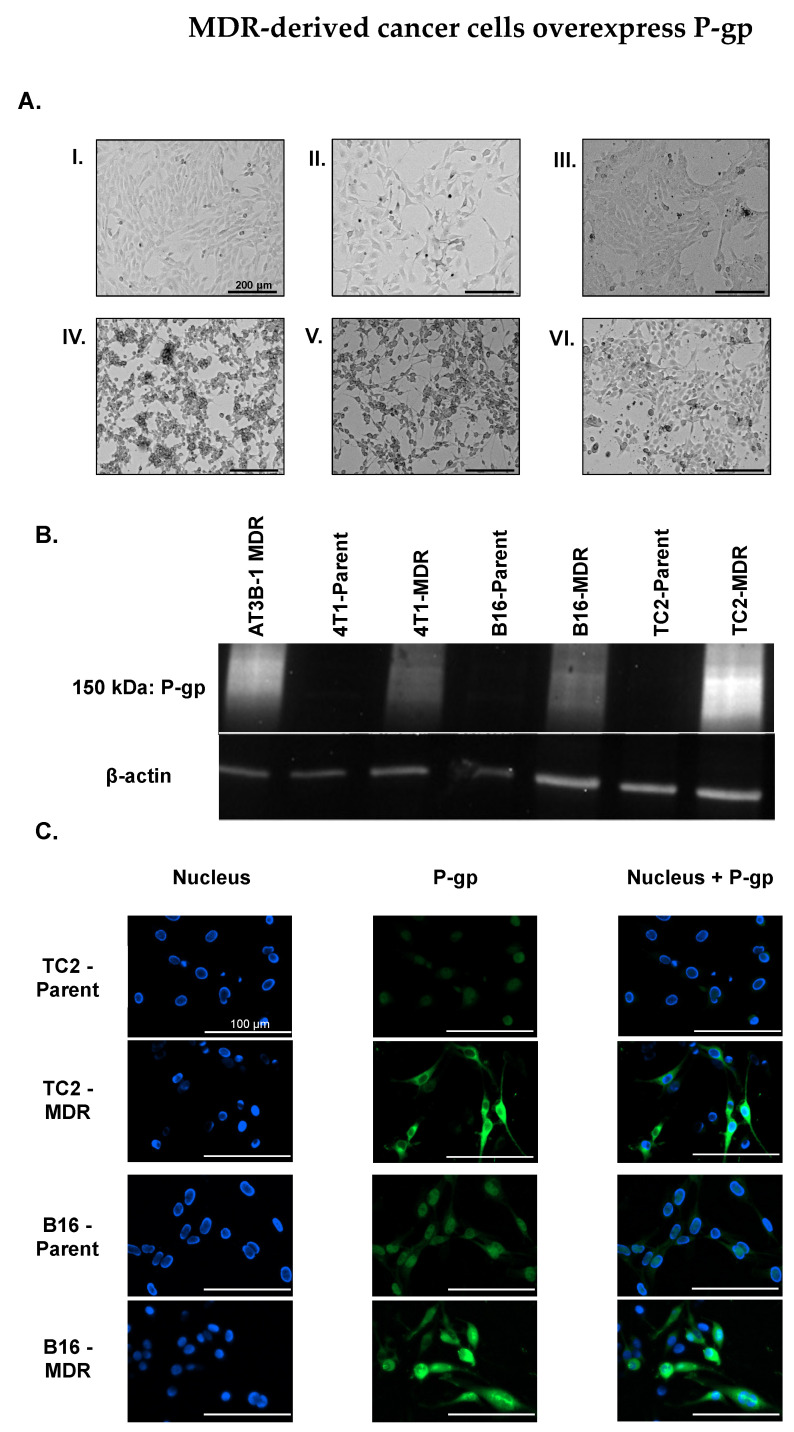
(**A**) MDR cancer cells were derived from parent cancer cell lines by incrementally increasing the Dox concentration in growth media. (**I**) TRAMP-C2 (TC2) prostate parent cancer cell line. (**II**) B16-F10 (B16) melanoma parent cancer cell line. (**III**) 4T1-Luc2 (4T1) breast parent cancer cell line. (**IV**) TC2-MDR cancer cell line. (**V**) B16-MDR cancer cell line. (**VI**) 4T1-MDR cancer cell line. Scale: 200 µm (**B**) Western blot shows the increased expression of P-gp in MDR cancer cells compared to parent cancer cell lines. (**C**) TC2-Parent, TC2-MDR, B16-Parent, and B16-MDR stained with Anti-P-gp-Alexa Fluor 488 antibody conjugate reveals the increased expression of P-gp in MDR-derived cancer cell lines, as well as membrane localization of P-gp.

MDR cancer cell lines were derived from parent cancer cell lines by introducing 1 nM Doxorubicin (Dox) in the growth media and doubling the concentration at each passage until cell populations capable of stable proliferation in 1 µM Dox were obtained (Figure 3A; Supplementary Material for MDR progression). This process is established to provide epigenetic pressure that increases expression of P-gp [38]. Western blot (Figure 3B) confirmed that MDR-derived cell lines expressed more P-gp than their parent cancer cell line counterparts with the amount of P-gp quantified in each of the MDR-derived cell lines (Figure S7). P-gp localization on cellular membranes was confirmed by fluorescence microscopy using an Anti-P-gp-Alexa Fluor 488 antibody conjugate (Figure 3C). The 4T1 cell lines were not used in the immunofluorescence assay due to spectral overlap between the antibody and GFP expression linked to the Luc2 gene.

### 2.3. Imiquimod Competes with Rhodamine 123 Efflux in MDR-Derived Cancer Cell Lines

**Figure 4:**
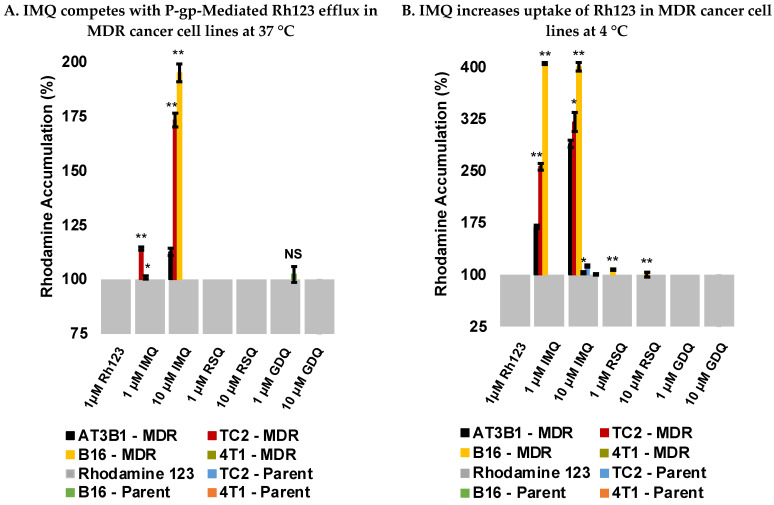
(**A**) Competitive experiments performed at 37 °C with P-gp substrate Rh123. IMQ leads to an increase in Rh123 accumulation in MDR-derived cancer cells under active transport conditions. Data representative of triplicate experiments. (**B**) Uptake experiments performed at 4 °C with P-gp substrate, Rhodamine 123. IMQ leads to an increase in Rh123 accumulation in MDR-derived cancer cells under passive diffusion conditions. Data representative of triplicate experiments. *p*-values were calculated between parent vs. MDR cancer cell type with * *p* < 0.01, ** *p* < 0.001 and NS: Not significant.

To determine whether the imidazoquinolines compete with known P-gp substrates, competitive efflux studies were performed with Rhodamine 123 (Rh123) under active transport conditions (37 °C) in both parent and MDR cancer cell lines. P-gp-mediated Rh123 efflux was confirmed by increased Rh123 retention, quantified by Mean Fluorescence Intensity (MFI), upon co-incubation with P-gp substrate Verapamil (VER) [39,40] and Tariquidar (TQR), a third-generation inhibitor of P-gp [41] (Figure S1). Significant increases in Rh123 retention were also observed in the MDR cell lines, particularly AT3B-1, TC2-MDR, and B16-MDR cells dually loaded with Rh123 and IMQ, compared to Rh123 alone (Figure 4A and Figure S5). No significant increase in Rh123 retention was observed with RSQ and GDQ, which could be indicative of either their lowered affinity for P-gp or decreased intracellular loading concentration relative to IMQ, which we subsequently determined by HPLC. None of the imidazoquinolines affected Rh123 retention for the 4T1-MDR or any parent cancer cell lines (Figure 4A). Additionally, there was no increase in fluorescence upon co-incubation with VER or TQR in the parent cell lines. These results were consistent with enhanced P-gp expression in the MDR cell lines promoting Rh123 and IMQ efflux. Interestingly, significant increases in MFI were observed in the presence of IMQ, in contrast to other studies reporting imidazoquinolines such as RSQ do not affect efflux [35]. From our competitive efflux experiments, we concluded that, with the exception of inconclusive results in 4T1-MDR cells, IMQ competes with Rh123 in the MDR cancer cell lines for P-gp-mediated efflux.

### 2.4. Imiquimod Increases Rhodamine 123 Uptake under Passive Diffusion Conditions

Although active transport efflux experiments confirmed that IMQ competes with Rh123 in most of the MDR cancer cell lines, but not in parent cancer cell lines, this trend was not observed with RSQ or GDQ. In order to determine if changing the loading conditions would impact the results observed in the competitive efflux experiments, uptake studies with Rh123 were performed under passive diffusion conditions (4 °C) where P-gp is inactive [42]. No significant increases in Rh123 accumulation were observed in most of the cell lines tested with RSQ and GDQ relative to Rh123 alone except for minimal differential accumulation with RSQ in B16-MDR cells (Figure 4B and Figure S6). VER exhibited the same trend as IMQ, as did TQR, though to a lesser extent (Figure S2). Similar to the competitive efflux experiments, this trend was only observed in the same MDR-cell lines, suggesting that IMQ leads to more Rh123 uptake in the MDR-derived cell lines compared to their parent counterparts under passive diffusion conditions.

### 2.5. Resiquimod and Gardiquimod Are Not Passively Taken up by MDR Cancer Cells

**Figure 5:**
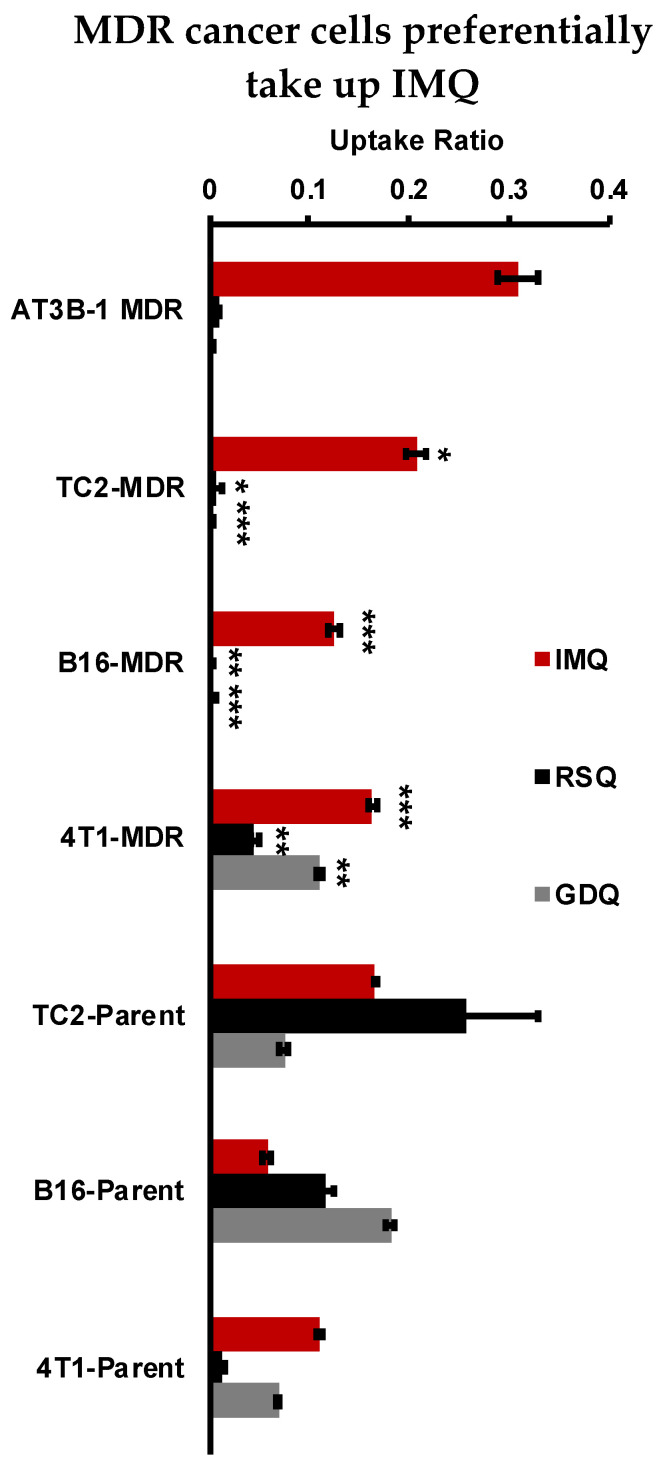
Uptake experiments performed by loading IMQ, RSQ, or GDQ (100 μM) under passive diffusion conditions in parent and MDR-derived cancer cell lines followed by analysis of imidazoquinoline in cell lysate by HPLC. Cellular uptake ratio was calculated by dividing the peak area of substrate in lysate by the peak area calibrated for 100 µM imidazoquinoline using standard solutions. Data representative of experiments performed in triplicate. *p*-values were calculated between parent vs. MDR cancer cell lines with * *p <* 0.05, ** *p* < 0.01, and *** *p* < 0.001.

To examine the effect of MDR on uptake, cell lysate was examined for imidazoquinolines following loading under passive conditions (4 °C). Cancer cells loaded with imidazoquinolines (100 µM) were lysed and lysate imidazoquinoline content was quantified by HPLC. Results of this experiment indicated that RSQ and GDQ were not significantly taken up by TC2-MDR, B16-MDR, or AT3B-1-MDR cells; however, IMQ showed efficient uptake under identical loading conditions (Figure 5). TC2-MDR cells had the highest intracellular IMQ (20.75 µM) among the MDR lines derived in-house. In comparison, all imidazoquinolines were taken up by the parent cancer cell lines. Here, the TC2-Parent (16.58 µM) had the highest IMQ uptake, followed by the 4T1-Parent (11.06 µM) and B16-Parent (5.72 µM) cell lines. RSQ was only detectable in parent cancer cell lines: TC2-Parent (25.84 µM), B16-Parent (11.65 µM), and 4T1-Parent (1.34 µM). Likewise, GDQ was only detected in parent cancer cell lines, with B16-Parent having the highest uptake (16.42 µM). Interestingly, detectable amounts of IMQ (16.31 µM), RSQ (4.47 µM), and GDQ (10.13 µM) are taken up by the 4T1-MDR-derived cancer cell line; however, significant competition for P-gp-mediated efflux was not observed with Rh123 for RSQ and GDQ. Furthermore, we also observed that MDR cell lines take up more IMQ compared to the parent cancer cells. Finally, because imidazoquinolines are also known to directly induce apoptosis in a variety of cancers, independent of immunogenic effects [43], we also confirmed that the tested concentrations/incubation times were not cytotoxic to the cancer cells via resazurin cell viability assay (Figure S4).

## 3. Discussion

Multidrug resistance is a major challenge for traditional chemotherapy, and attributed to many different mechanisms, including increased DNA damage repair, reduced apoptosis, aberrant drug metabolism, and enhanced drug efflux [44,45]. The cancer cell types chosen for this study were B16-F10 (B16) melanoma, TRAMP-C2 (TC2) prostate, and 4T1-Luc2 (4T1) breast cancer. These cell lines were primarily chosen based on their ability to acquire drug resistance [46,47,48], as well as the established efficacy of TLR 7/8 agonists in corresponding in vivo models [26,49,50,51]. As a positive control we chose AT3B-1 prostate cancer cells, as opposed to Caco-2 or MDCK-II lines routinely used for measuring efflux of small molecules [52], because AT3B-1 cells possessed the previously well-characterized Dox-derived MDR phenotype [53] and have subsequently been used in studies involving membrane transport through P-gp [54].

The imidazoquinoline immunostimulants chosen for this study were: Imiquimod (IMQ), Resiquimod (RSQ), and Gardiquimod (GDQ). We chose these particular imidazoquinolines for their extensive use in cancer immunotherapy. In particular, the TLR 7 agonist IMQ is FDA-approved for treatment of basal skin cell carcinoma and known to confer anti-cancer immunogenicity [55]. RSQ, a more potent TLR 7/8 dual agonist, features nanomolar potency [56], and is capable of activating TLR 8 in humans which is expressed by myeloid-derived dendritic cells [57,58], an advantage when compared to IMQ. GDQ, a TLR 7 agonist, is more potent than IMQ, and likewise exhibits enhanced antitumor effects [59].

Previously, our own work implied that IMQ [29] and RSQ [30] undergo drug efflux from a range of cancer cell lines. However, specific routes of efflux were only indirectly investigated. In the present study, we directly confirmed that IMQ, RSQ, and GDQ are substrates of P-gp, with variable affinity, using an ATPase membrane transport study (Figure 2). We also concluded that the less potent TLR 7 agonist IMQ is a better substrate for P-gp efflux than RSQ or GDQ in both membrane vesicles and MDR cancer cells.

Next, we created MDR cancer cells which overexpressed P-gp by growing the non-MDR Parent cell lines in increasing concentrations of Dox from 1 nM to 1 µM. Each of the cancer cell lines reached the 1 µM Dox threshold at different times. The TC2-MDR version took 3 months, the B16-MDR took 7 months, and the 4T1-MDR cells took over 8 months (see Supplementary Materials for timeline). Via brightfield microscopy (Figure 3A), we observed that cell morphology changed to elongated structures at increased Dox concentration, perhaps suggestive of the epithelial to mesenchymal transition that can occur upon chronic exposure to chemotherapeutics [60]. Regardless, our MDR cancer cell lines increased P-gp expression compared to parent cell lines, as quantified by Western blot (Figure 3B and Figure S7) with visually confirmed membrane localization via fluorescence microscopy (Figure 3C).

With both imidazoquinoline P-gp substrate specificity and P-gp expression in MDR cancer cell lines confirmed, we next investigated imidazoquinoline efflux in our parent and MDR cell lines. Here, IMQ competed with Rh123 for efflux in most of the MDR-cancer cell lines, a trend not observed in the parent cancer cell lines (Figure 4A and Figure S5). This result directly correlated with P-gp expression in the MDR-derived cancer cell lines and is consistent with our previous report that IMQ competes with Rh123 for efflux in AT3B-1 cells [29]. Alternatively, the baseline MFIs (loading with Rh123 alone) of MDR-derived cancer cells were lower than the parent cancer cell lines, as is expected with enhanced efflux potential from acquired drug resistance (Figure S5) [61]. This means a smaller absolute fluorescence could appear as a larger signal when reported as a percent of baseline accumulation. That said, it is possible that poor uptake of RSQ and GDQ in MDR cancer cell lines could be responsible for the lack of competition observed in the efflux experiments (Figure 4 and Figure S6). We also observed enhanced uptake of IMQ in MDR cells compared to parent cell lines, both with and without Rh123. This could be explained by a variety of mechanisms. For example, drug-resistant cells could have altered membrane permeability beyond transport protein expression. Another possibility is that P-gp may influence substrate influx as well [62].

Based upon these results, we conclude that P-gp efflux susceptibility, which correlates to hydrophobicity/cLogP, should be considered, alongside potency, when choosing the optimal TLR agonist for delivery to MDR cancers. cLogP can influence P-gp efflux and does not negatively impact passive permeability unless the values fall well outside drug-like ranges (cLogP < 1 or cLogP ≥ 7) [15]. IMQ falls within this range; however, RSQ and GDQ fall outside this range which could explain both the lack of uptake of RSQ and GDQ as well as their minimal competition for efflux.

It is important to note that even though expression of P-gp was increased in the 4T1-MDR cell line, relative to the non-MDR parent line, we did not observe imidazoquinoline competition with Rh123. Interestingly, we did observe an increase in Luc2 gene in the MDR-derived cell line, directly correlating to the acquisition of drug resistance (Figures S5 and S6). While this study demonstrates that imidazoquinolines, particularly IMQ, are substrates for P-gp mediated efflux, it is also likely that imidazoquinolines could serve as substrates for some of the many other ABC transporters, which may have led to no change in Rh123 accumulation, especially in the 4T1-MDR cell line. It is also possible that Dox-derived MDR provokes compensatory expression of other ABC transporters or other ABC-independent efflux mechanisms as well. Although some generalizable differences in substrate scopes do exist between transport proteins implicated in drug efflux, there is also significant overlap, particularly with proteins associated with MDR that further complicate both development and analysis of efflux [63,64,65].

## 4. Materials and Methods

### 4.1. Materials

The B16-F10 melanoma cell line was purchased from the American Type Culture Collection (ATCC, Manassas, VA, USA). As per manufacturer instructions, the B16-F10 cell line (ATCC, Manassas, VA, USA, CRL-6475, mouse melanoma) was grown in complete culture media composed of DMEM (VWR, Radnor, PA, USA, 6777-406) with 4.5 g L^−1^ glucose, 2 mM L-glutamine, 100 U mL^−1^ PenStrep (Caisson Labs, Smithfield, VA, USA, PSL01), and supplemented with 10% Premium Grade HI-FBS (VWR, Radnor, PA, USA, 97068-091). Media was changed every 3–4 days. Cells were passaged upon reaching 80% confluence. Trypsin solution (Sigma Aldrich, St. Louis, MO, USA, T-4049) was used per manufacturer instructions to release cells before passaging which involved changing media, counting, and seeding 3 × 10^5^ cells in 35 mL of new complete media in a new T-175 culture flask (VWR, Radnor, PA, USA, 10861-650).

The TRAMP-C2 prostate cell line was purchased from ATCC. As per manufacturer instructions, the TRAMP C2 cell line (ATCC, Manassas, VA, USA, CRL-2731, mouse transgenic adenocarcinoma) was grown in complete culture media composed of DMEM with 4.5 g L^−1^ glucose, 2 mM L-glutamine, 100 U mL^−1^ PenStrep, and supplemented with 1 µg mL^−1^ Insulin (Sigma-Aldrich, St. Louis, MO, USA, I0516), 2 nM (+)-Dehydroisoandrosterone (VWR, Radnor, PA, USA, 200008-124), 5% HI-FBS, and 5% Nu-Serum IV (Corning, Corning, NY, USA, 355504). Media was changed every 3–4 days. Cells were passaged upon reaching 80% confluence. Trypsin solution (Sigma Aldrich, St. Louis, MO, USA, T-4049) was used per manufacturer instructions to release cells before passaging which involved changing media, counting, and seeding 3 × 10^5^ cells in 35 mL of new complete media in a new T-175 culture flask (VWR, Radnor, PA, USA, 10861-650).

4T1-Luc2 breast cancer cell line was gifted from Darrell Irvine’s lab (Massachusetts Institute of Technology, Cambridge, MA, USA). The 4T1-Luc2 cell line (ATCC, Manassas, VA, USA, CRL-2539-LUC2, mouse mammary gland carcinoma) was grown in complete culture media composed of DMEM with 4.5 g L^−1^ glucose, 2 mM L-glutamine, 100 U mL^−1^ PenStrep, and supplemented with 10% HI-FBS. Media was changed every 3–4 days. Cells were passaged upon reaching 80% confluence. Trypsin solution (Sigma Aldrich, St. Louis, MO, USA, T-4049) was used per manufacturer instructions to release cells before passaging which involved changing media, counting, and seeding 3 × 10^5^ cells in 35 mL of new complete media in a new T-175 culture flask (VWR, Radnor, PA, USA, 10861-650).

AT3B-1 prostate cancer cells were chosen for their well-characterized P-gp expression as a result of epigenetic pressure caused by exposure to the Doxorubicin [53]. The AT3B-1 cell line was purchased from ATCC. The AT3B-1 cell line (ATCC, Manassas, VA, USA, CRL-2375, rat MDR prostate carcinoma) was grown in complete culture media composed of DMEM with 4.5 g L^−1^ glucose, 2 mM L-glutamine, 100 U mL^−1^ PenStrep, supplemented with 10% HI-FBS and 1 μM Doxorubicin (TCI America, Portland, OR, USA, D4193100MG). Media was changed every 3–4 days. Cells were passaged upon reaching 80% confluence. Trypsin solution (Sigma Aldrich, St. Louis, MO, USA, T-4049) was used per manufacturer instructions to release cells before passaging which involved changing media, counting, and seeding 3 × 10^5^ cells in 35 mL of new complete media in a new T-175 culture flask (VWR, Radnor, PA, USA, 10861-650).

The B16-F10 Melanoma (B16), TRAMP C-2 prostate (TC2), and 4T1-Luc2 breast (4T1) parent cancer cell lines were seeded at 3 × 10^5^ cells in T-175 culture flasks separate from the parent cancer cell lines. These cells were cultured in complete cell media which contained Doxorubicin (Dox). The original media was composed of: DMEM with 4.5 g L^−1^ glucose, 2 mM L-glutamine, 100 U mL^−1^ PenStrep, 10% HI-FBS and 1 nM Dox. The media was changed every 3–4 days until the cells reached 80% confluence. Trypsin solution (Sigma Aldrich, St. Louis, MO, USA, T-4049) was used per manufacturer instructions to release cells before passaging which involved changing media, counting, and seeding 3 × 10^5^ cells in 35 mL of new complete media in a new T-175 culture flask (VWR, Radnor, PA, USA, 10861-650). Dox concentration was doubled only after stable proliferation, which for some cell lines took multiple passages, before reaching a final concentration of 1 μM (Figure 2).

### 4.2. ATPase Assay

Colorimetric measurement of imidazoquinoline interaction with P-gp was determined using a PREDEASY ATPase Assay Kit (SOLVO Biotechnology, Sigma-Aldrich, St. Louis, MO, USA) in 96-well plate format following the manufacturer’s protocol. Stock solutions of developer and blocker were diluted using Ultrapure DNase/Rnase Free Distilled Water (Invitrogen, Waltham, MA, USA, 10977015). Briefly, across individual wells, Imiquimod (IMQ) (eNovation Chemicals, Green Brook, NJ, USA, SY017571), Resiquimod (RSQ) (Accel Pharmtech, East Brunswick, NJ, USA, XP2356), Gardiquimod (GDQ) (synthesized in-house; see Supplementary Materials) were added at 8 different concentrations (1–200 μM) to membrane vesicles expressing hMDR1. Each well contained 4 µg membrane protein, and 1 µL of imidazoquinoline was added to arrive at the final concentrations noted. The plate was pre-incubated (37 °C, 10 min) before 10 µL of MgATP solution was added to start the reaction. The plate was incubated (37 °C, 10 min) before the ATPase reaction was quenched using 100 µL of Developer Solution at room temperature. After 2 min, 100 µL of Blocker solution was added to each well at room temperature before incubation (37 °C, 30 min). Following incubation, the absorbance was measured using a microplate reader at 610 nm. Absorbance values were used to calculate liberated P_i_ (Figure 2) and K_D_.

### 4.3. Western Blot

Cell lysates were extracted using Triton X-100 lysing buffer, and lysate was quantified using Pierce BCA Protein Assay Kit (ThermoFisher Scientific, Waltham, MA, USA, 23225). For each cell lysate, 20 μg of protein was run on a 4–15% SDS gel (Bio-Rad, Hercules, CA, USA, 4561083DC) and electrotransferred onto a PVDF membrane. The membrane was washed with TBS and blocked overnight with 3% BSA in TBST. The membrane was incubated with primary Rabbit anti-P-gp antibody (Abcam, Waltham, MA, USA, ab170904) for 2 h, washed 2× for 10 min with TBST, and incubated with a secondary Goat anti-Rabbit IgG HandL (Abcam, Waltham, MA, USA, ab97051) antibody for 1 h. Rabbit anti-β-actin antibody (Abcam, Waltham, MA, USA, ab8227) along with the MDR AT3B-1 cell line were used as controls (Figure 3B). As per manufacturer’s instructions, the primary antibody detects the predominant protein band migrating in the region of 180–200 kDa and typically will demonstrate a smear on the membrane in the region of 150–300 kDa due to the glycosylation profile of the protein [66].

### 4.4. Immunofluorescence Assay

Cancer cells (parent and MDR-derived) were plated on glass coverslips (neuVitro, Vancouver, WA, USA, GG-25-1.5-pdl) in a 6-well plate and allowed to reach 70% confluency. The cells were fixed using 4% formaldehyde for 15 min at room temperature. After three washes with 1 mL PBS, the cells were blocked (10% HI-FBS in PBS) for 1 h at 37 °C. Cells were incubated with Mouse anti-P-gp antibody conjugated to Alexa Fluor 488 (Santa Cruz Biotechnology, Dallas, TX, USA, sc55510 AF488) overnight in the dark at 4 °C before incubation with Mouse IgG Fc binding protein conjugated to CruzFluor 488 (Santa Cruz Biotechnology, Dallas, TX, USA, sc533653) in the dark at room temperature for 1 h. Finally, coverslips were washed in PBS and mounted with Vectashield antifade mounting medium with DAPI (Vector Labs, Burlingame, CA, USA, H-2000-10) diluted in Vectashield antifade mounting media (Vector Labs, Burlingame, CA, USA, H-1900-10) to 0.1 μg mL^−1^. Fluorescent images (40×) were acquired on a Lionheart FX (BioTek Instruments) microscope (Figure 3C).

### 4.5. Competitive Efflux Studies with Rhodamine 123

Cancer cells (parent and MDR-derived) were passaged, and 1 × 10^6^ cells were used for each compound tested with Rhodamine 123 (Rh123) (Cayman Chemical, Ann Arbor, MI, USA, 16672). Cells were suspended in DMEM, supplemented with 10% HI-FBS, and 1 μM Rh123; and incubated (37 °C, 30 min). Following incubation with Rh123, 1 or 10 μM of imidazoquinoline (IMQ, RSQ, GDQ), and/or P-gp inhibitor of Verapamil (VER) (Cayman Chemical, Ann Arbor, MI, USA, 14288), or Tariquidar (TQR) (Sigma-Aldrich, St. Louis, MO, USA, SML 1790) were added to the cells and incubated for another 30 min at 37 °C. After incubation, samples were centrifuged (300 RCF, 5 min) and the supernatant discarded. Cells were fixed with 4% formaldehyde for 15 min at room temperature. The samples were centrifuged (300 RCF, 5 min) and suspended in 1 mL cold FACS buffer. Finally, the Mean Fluorescence Intensity (MFI) of Rh123 in the samples was measured via flow cytometry (BD Accuri C6 Plus). Results are representative of triplicate experiments and normalized to MFI for 1 μM Rh123 (Figure 4A and Figure S5; see Supplementary Materials for histograms).

### 4.6. Uptake Studies with Rhodamine 123

Cancer cells (parent and MDR-derived) were passaged, and 1 × 10^6^ cells were used for each compound being tested with Rh123. Cancer cells were suspended in DMEM, supplemented with 10% HI-FBS, and 1 μM Rh123. The cells were incubated (4 °C, 30 min) followed by adding IMQ, RSQ, GDQ, VER, or TQR (1 or 10 μM) and incubating further for 30 min at 4 °C. Next, samples were centrifuged (300 RCF, 5 min) and the supernatant discarded. Cells were fixed with 4% formaldehyde for 15 min at room temperature. The samples were centrifuged again (300 RCF, 5 min), suspended in 1 mL ice cold FACS buffer, and analyzed in triplicate for Rh123 MFI via flow cytometry. Samples were normalized to uptake of 1 μM Rh123 as indicated in (Figure 4B and Figure S6; see Supplementary Materials for histograms).

### 4.7. Cellular Uptake Studies

Cancer cells (parent and MDR-derived) were passaged, and 5 × 10^6^ cells were used for each experiment. Cells were suspended in 1 mL DMEM, supplemented with 10% HI-FBS, with 100 μM of IMQ, RSQ, or GDQ, and incubated at 4 °C for 30 min. Samples were then centrifuged (300 RCF, 5 min) and washed 2 times with 1 mL cold PBS. The samples were re-suspended in lysing buffer: For IMQ and RSQ, lysing buffer consisted of 40% HPLC Grade acetonitrile (ACN) in HPLC Grade water (H_2_O) with 0.1% Trifluoroacetic acid (TFA) and 1% *v*/*v* Trition X-100. Due to solubility differences, GDQ lysing buffer consisted of HPLC Grade water with 0.1% TFA and 1% *v*/*v* Triton X-100. Cells were lysed on ice for 20 min. Following the lysing step, samples were centrifuged (12500 RCF, 10 min) with the supernatant collected and filtered using a 0.2 μm PTFE filter. Filtered samples were analyzed by HPLC (Thermo Dionex UltiMate 3000 running Chromeleon software (V6.80 SR14) with a C18 analytical column (Phenomenex XB-C18 100Å, 250 × 4.6 mm, 5 µm) at a flow rate of 1 mL/min, A: H_2_O with 0.1% TFA, B: ACN with 0.1% TFA, with UV detection at 254 nm. For IMQ and RSQ an isocratic method (40% B) was used. For GDQ, a gradient method (10% B for 5 min, 10 to 95% B over 14 min, 95% B for 10 min) was used. For each of the imidazoquinolines, 9-point calibration curves were derived using standards with concentrations ranging from 500 nM to 250 μM. The calibration curves were used to fit using linear regression for each imidazoquinoline: IMQ (y = 0.039x; R^2^ = 0.9976), RSQ (y = 0.0447x; R^2^ = 0.9994), and GDQ (y = 0.0271x; R^2^ = 0.9996). All samples were analyzed in triplicate. Cellular uptake ratio was obtained by dividing the peak area of substrate in lysate by the peak area calibrated for 100 μM imidazoquinoline (Figure 5).

### 4.8. Cytotoxicity Assay

Resazurin Cell Viability Assay kit (Abcam, Waltham, MA, USA, ab129732) was used to determine if the concentrations of IMQ, RSQ, and GDQ used in experiments were cytotoxic to the cancer cell lines under experimental conditions. To begin, cancer cells were plated in complete cell media (DMEM, 10% HI-FBS) in 96-well plates, with two rows per plate of each density/well tested: 2000 cells/well, 25,000 cells/well, 50,000 cells/well, and 100,000 cells/well. Cells were treated with 20 μL of 1 mM solutions of IMQ, RSQ, and GDQ, to give a final concentration per well of 100 μM for each compound. Plates were incubated at 37 °C for 1 h. After incubation, 10 μL of resazurin stain was added to wells in alternating rows on the plate, such that for each density and compound tested in triplicate there was a corresponding blank without stain added. Plates were incubated further with absorbance measured at 570 nm and 600 nm at 1, 2, 3, 4, and 24 h intervals. The absorbance measurements at 600 nm, which correlated to the absorbance of resazurin, were subtracted from those taken at 570 nm, correlating to the resorufin absorbance. After this, values were normalized to cell viability of the negative control. Experiments were performed in triplicate and the 3 h time-point for 5 × 10^5^ cells is shown (Figure S4).

## 5. Conclusions

In conclusion, this study demonstrates the imidazoquinolines IMQ, RSQ, and GDQ are substrates for P-gp and begins to elucidate differences in their trafficking in cancer cells as a consequence of acquired drug resistance. Using Dox to derive MDR-versions of B16, TC2, and 4T1 cells resulted in enhanced P-gp expression and IMQ efflux. Additionally, using competitive experiments with Rh123, we demonstrate that IMQ competes with Rh123 P-gp efflux in the MDR phenotypes. Ultimately, this work contributes to the development of new cancer immunotherapies, particularly imidazoquinoline prodrugs, which could be enhanced by means of drug efflux following intracellular liberation of active drug. We believe this work that begins to examine imidazoquinoline trafficking will prove useful in the future rational design of immunotherapeutics with enhanced susceptibility to P-gp efflux that enable increased bioavailability, in MDR cancers.

## 6. Patents

A.J.B., A.E.N., and R.J.M. are inventors on WSU’s patent 11,117,918; A.E.N. and R.J.M. are owners of Astante Therapeutics Inc. both of which use concepts related to those in this work.

## Figures and Tables

**Table 1:** cLogP and K_D_ values of imidazoquinolines. cLogP was calculated using ChemDraw 19.1 Software—PerkinElmer. K_D_ was estimated (sum of squares) from the membrane vesicle P-gp assay.

Imidazoquinoline	cLogP	K_D_ (μM)
Imiquimod	1.428	7.66
Resiquimod	0.036	24.37
Gardiquimod	−0.254	N.D.

## Data Availability

Data is contained within the article or Supplementary Material.

## Supplementary Materials

The following are available online at https://www.mdpi.com/article/10.3390/ph14121292/s1, Figure S1: Verapamil (VER) and Tariquidar (TQR) compete with Rhodamine 123 (Rh123) in all cancer cells, leading to more accumulation in MDR cancer cell lines at 37 °C, Figure S2: Verapamil (VER) and Tariquidar (TQR) lead to an increased uptake of Rhodamine 123 (Rh123) in MDR-derived cancer cells at 4 °C, Figure S3: Imidazoquinolines do not inhibit maximal vanadate sensitive ATPase activity, Figure S4: Imidazoquinolines are not cytotoxic to cancer cells at high concentrations, Figure S5: Mean Fluorescence Intensities of competitive experiments with Rhodamine 123 at 37 °C, Figure S6: Mean Fluorescence Intensities of uptake experiments with Rhodamine 123 at 4 °C, Figure S7: Amount of P-gp present in the MDR cancer cells as per the Western blot. Additionally, K_D_ calculations and values of imidazoquinolines, flow cytometry histograms, microscope images of the progression of parent to MDR-derived cancer cell lines, and synthetic procedure and characterization of GDQ [67,68,69,70] can also be found in the supporting information.
