# Supplementary material for: Acquired Drug Resistance Enhances Imidazoquinoline Efflux by P-Glycoprotein
Source: Pharmaceuticals (Basel). 2021 Dec 10;14(12):1292. doi: 10.3390/ph14121292 (PMC8705394; doi:10.3390/ph14121292)
Supplement: Supplementary file 1 [file pharmaceuticals-14-01292-s001.zip › pharmaceuticals-1475344-supplementary.pdf]

Acquired drug resistance enhances imidazoquinoline efflux by P-glycoprotein

Anunay J. Pulukuri, Anthony J. Burt, Larissa K. Opp, Colin M. McDowell, Maryam Davaritouchaee, Amy E. Nielsen, and Rock J. Mancini

Washington State University, Department of Chemistry

1470 E College Ave, Pullman, WA 99164, United States

Table of Contents:

K_D values of Imidazoquinolines 2

Supporting Figures 3

Progression of MDR derived cancer cell lines 10

Flow Cytometry Histograms from Competitive Experiments 13

Flow Cytometry Histograms from Uptake Experiments 14

Synthetic Procedure for Gardiquimod 15

Characterization of Gardiquimod 17

References 23

K_D values of imidazoquinolines

$$\frac{P_i}{P_i\,max} = \frac{[imidazoquinoline]}{[imidazoquinoline] + K_D}$$

Imidazoquinoline	Calculated K_D value (µM)	Sum of squares
Imiquimod (IMQ)	7.66	0.01999
Resiquimod (RSQ)	24.37	0.14159
Gardiquimod (GDQ)	-	-

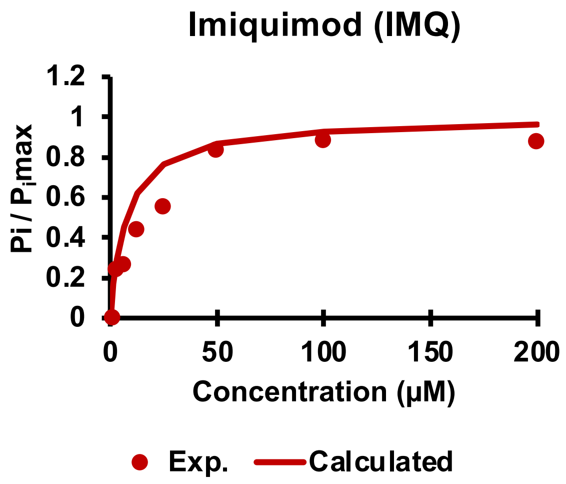

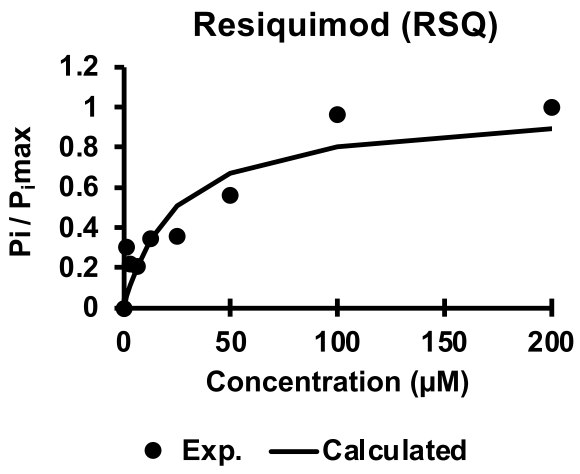

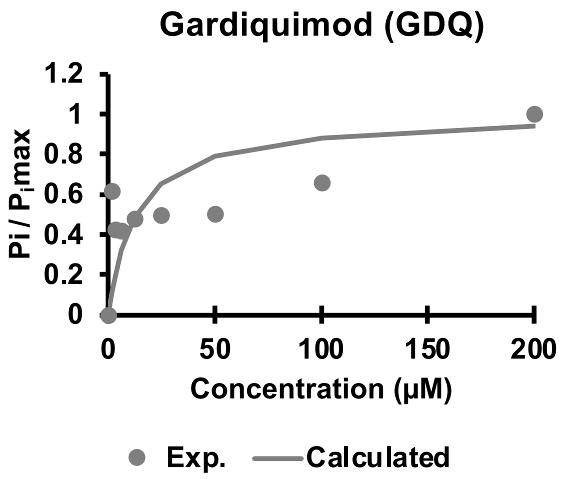

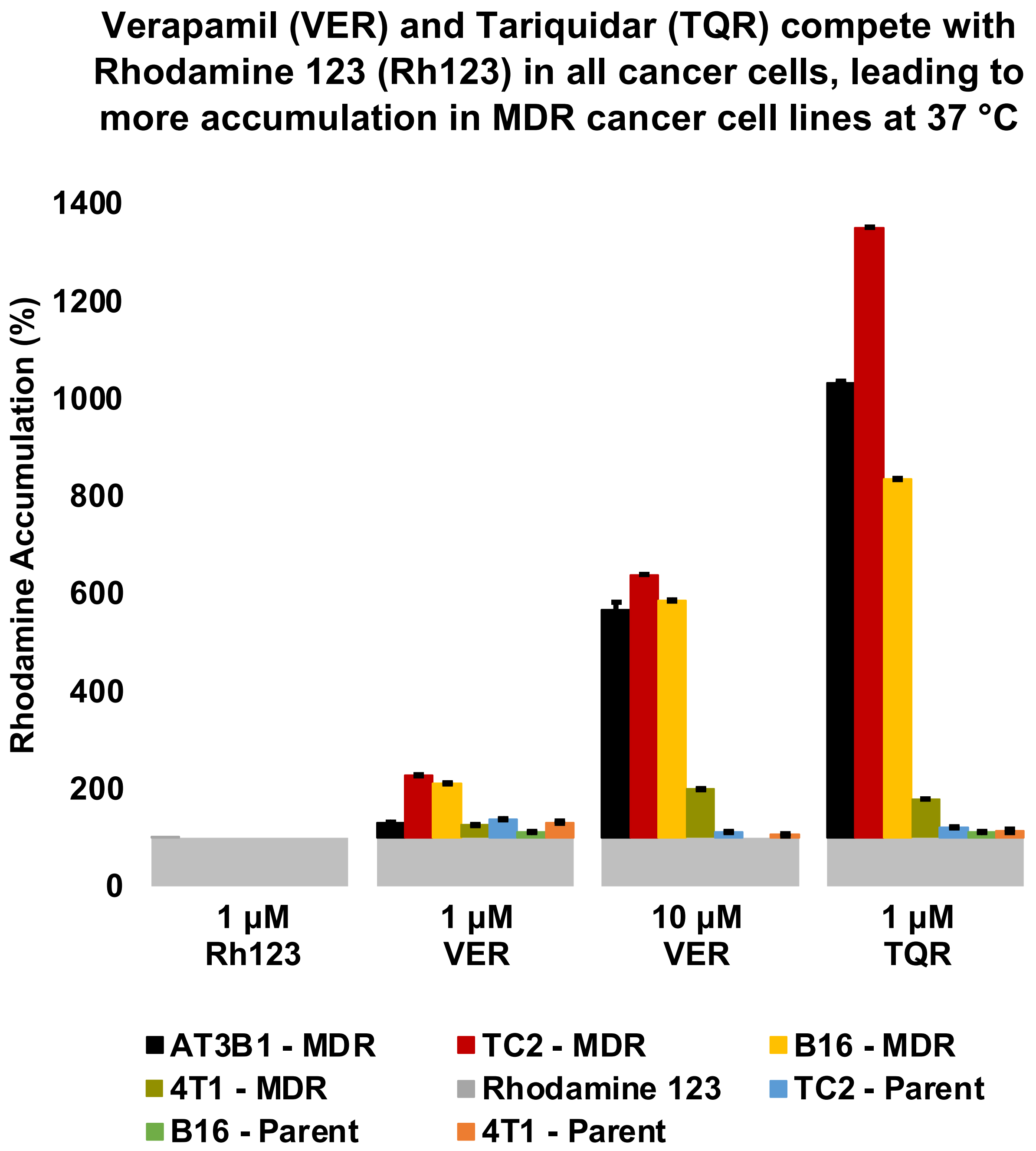

Figure S1: Competitive experiments with a known fluorescent substrate of P-gp, Rhodamine 123 (Rh123), in the presence of Verapamil (VER), a known competitive substrate and a first-generation inhibitor, and Tariquidar (TQR), a third-generation inhibitor. VER and TQR compete with Rh123 in all of the cell lines tested. However, more Rh123 accumulation is observed in the MDR-derived cancer cell lines as compared to the parent cancer cell lines. Data representative of experiments repeated in triplicate.

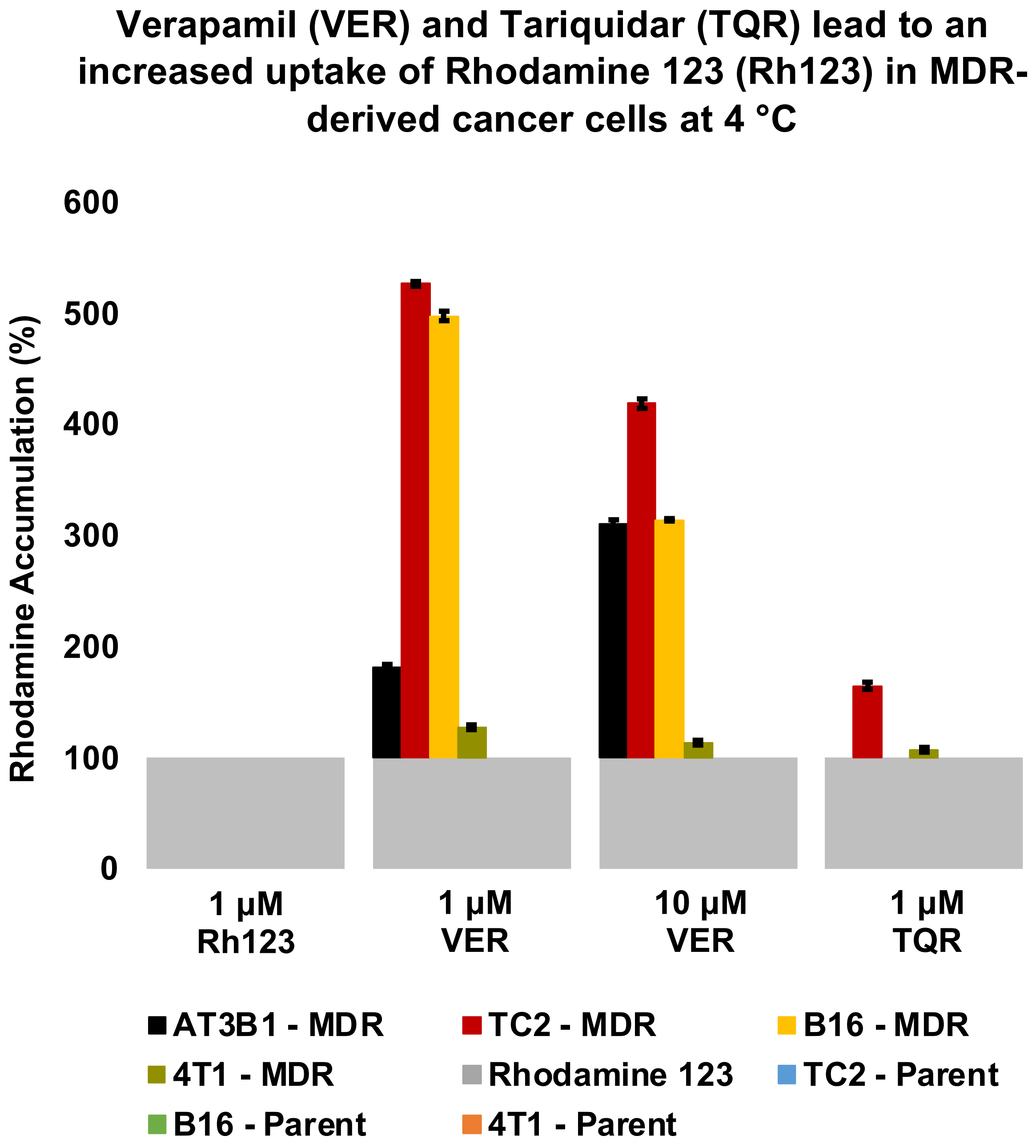

Figure S2: Uptake experiments with a known fluorescent substrate of P-gp, Rhodamine 123 (Rh123), in the presence of Verapamil (VER), a known competitive substrate and a first-generation inhibitor, and Tariquidar (TQR), a third-generation inhibitor. VER and TQR lead to an increased uptake of Rh123 under passive diffusion conditions in MDR-derived cancer cell lines. Data representative of experiments repeated in triplicate.

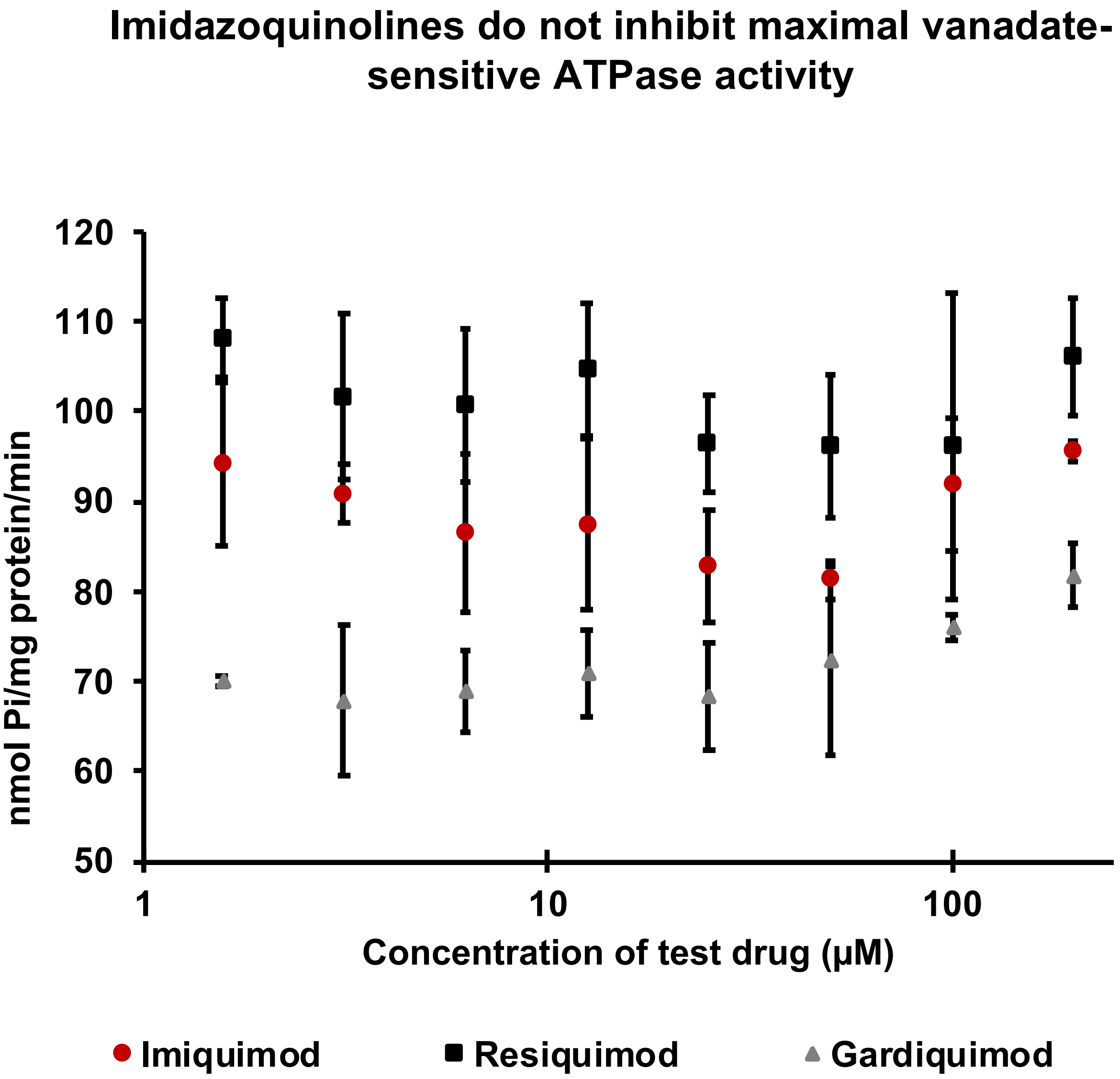

Figure S3: IMQ, RSQ, and GDQ do not inhibit maximal vanadate-sensitive ATPase activity in the inhibition test, a complementary test from the ATPase assay. As per manufacturer's instructions, the inhibition test is carried out in the presence a known strong activator of the transporter. Slowly transported compounds may inhibit maximal vanadate-sensitive ATPase activity and interactions detected indicate the interaction of the test compound with the transporter, but do not give information on the nature (substrate or inhibitor) of the interaction.

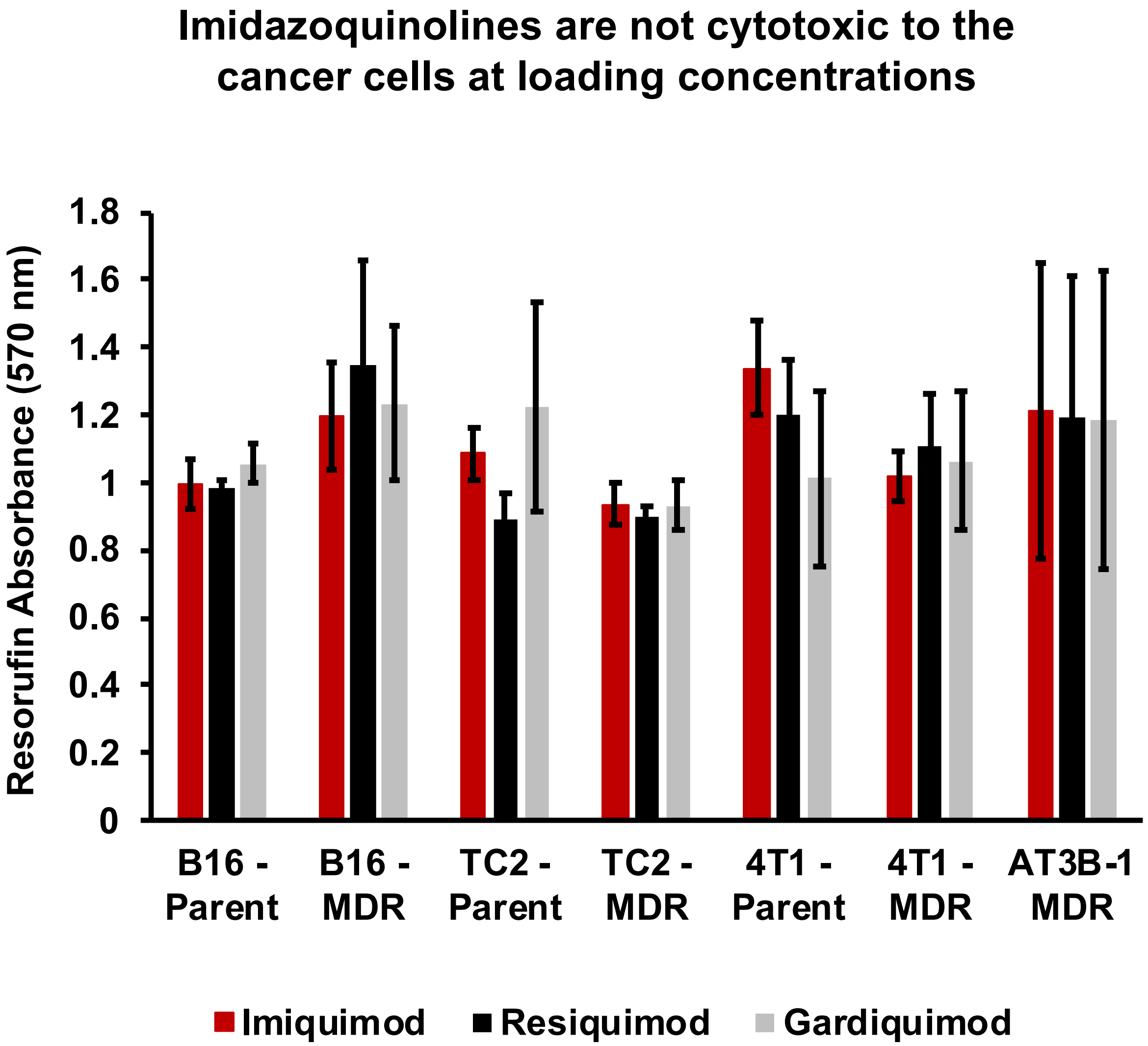

Figure S4: Resazurin Cell Viability Assay kit (Abcam - ab129732) was used to determine if the concentrations of imidazoquinolines used in experiments were cytotoxic to the cell lines tested: B16, B16 MDR, TC2, TC2 MDR, 4T1, 4T1 MDR, and AT3B1. Cells were treated with 20uL of 1 mM solutions of imidazoquinoline in 5% DMSO in PBS to give a final concentration per well of 100 μM for each compound. The absorbance measurements at 600 nm, which correlate to the absorbance of resazurin, were subtracted from those taken at 570 nm, correlating to the resorufin absorbance. After this, the appropriate blanks were subtracted, giving a resulting absorbance measurement that correlated directly to the number of cells in each well. The data shown here represents a density of 50,000 cells/well with a 3h incubation time with stain.

Mean fluorescence intensities of competitive experiments with Rhodamine 123 at 37 °C

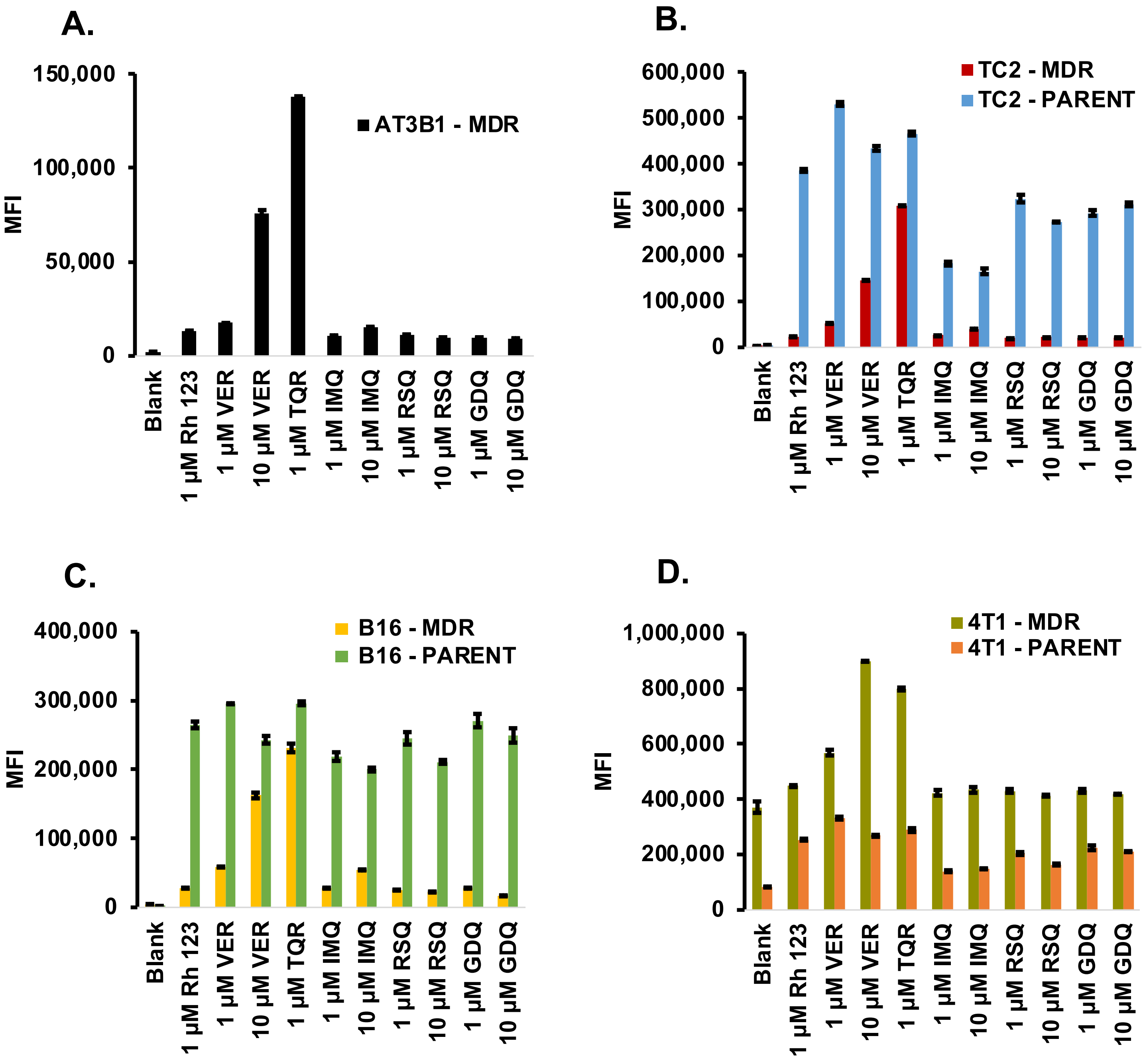

Figure S5: Raw Mean Fluorescence Intensities (MFI) acquired via FITC filter from the competitive experiments performed in the presence of Rhodamine 123 (Rh123). **A)** AT3B1 – MDR MFIs. **B)** TC2 – MDR vs. TC2 – Parent MFIs. **C)** B16 – MDR vs. B16 Parent MFIs. **D)** 4T1 – MDR vs. 4T1 – Parent MFIs.

Mean fluorescence intensities of uptake experiments with Rhodamine 123 at 4 °C

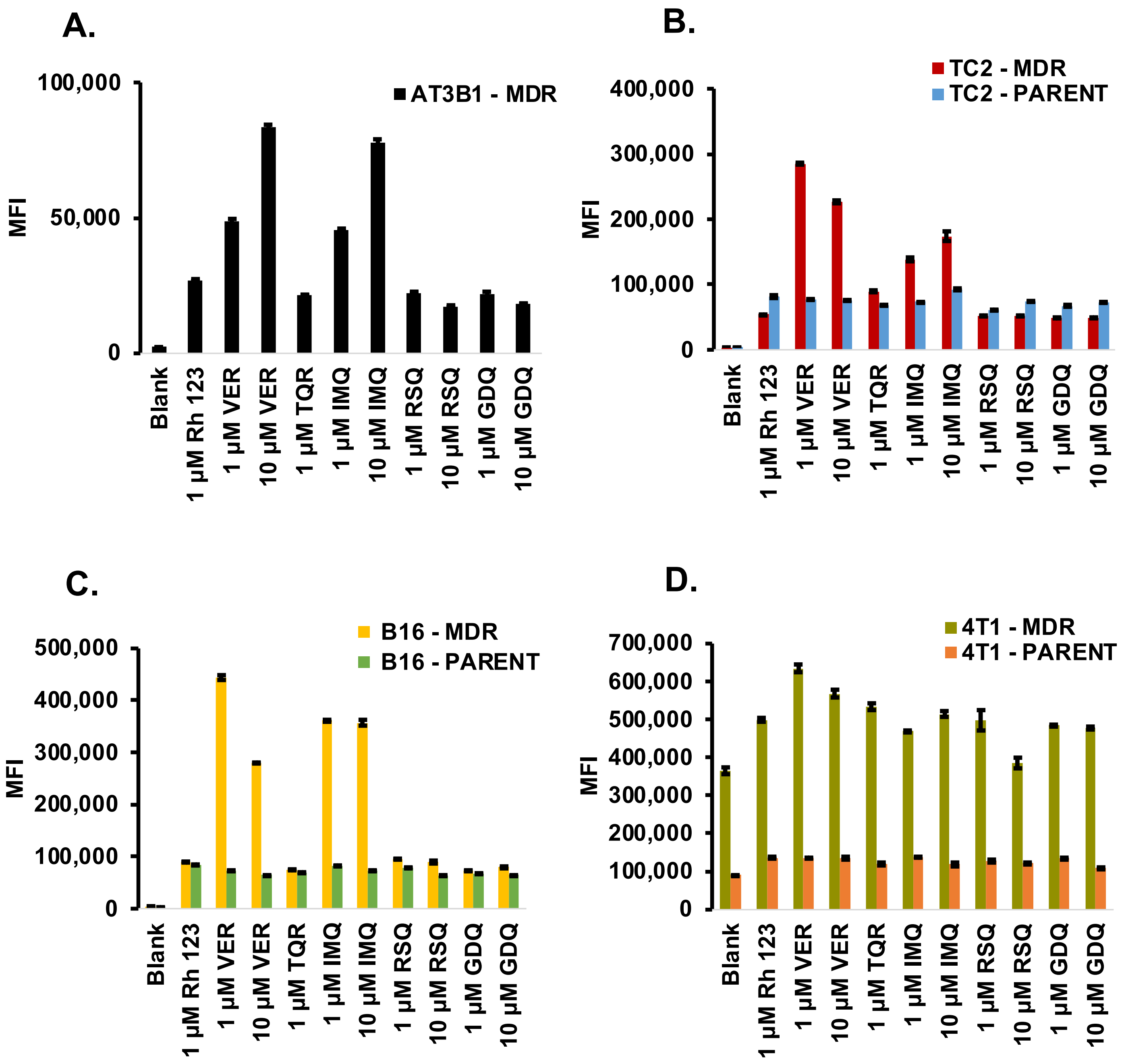

Figure S6: Raw Mean Fluorescence Intensities (MFI) acquired via FITC filter from the uptake experiments performed in the presence of Rhodamine 123 (Rh123). **A)** AT3B1 – MDR MFIs. **B)** TC2 – MDR vs. TC2 – Parent MFIs. **C)** B16 – MDR vs. B16 Parent MFIs. **D)** 4T1 – MDR vs. 4T1 – Parent MFIs.

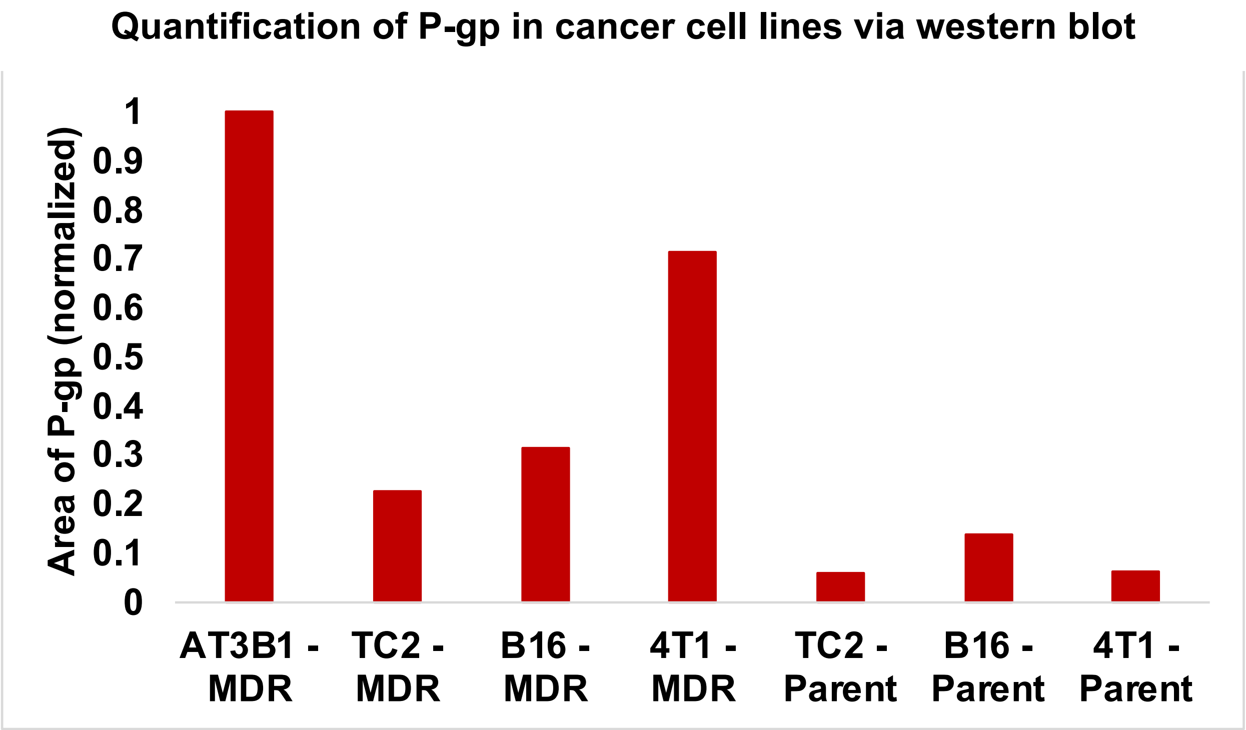

Figure S7: Area of P-gp in MDR-derived vs. Parent cancer cell lines as per our western blot normalized to the value of AT3B-1 MDR cancer cells (P-gp positive control), and β-actin (loading control).

Progression of TRAMP C2 MDR derived cancer cell line from TRAMP C2 Parent cancer cell line

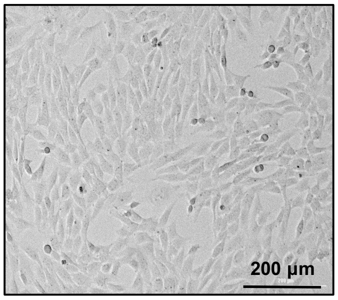

Parent TRAMP C2

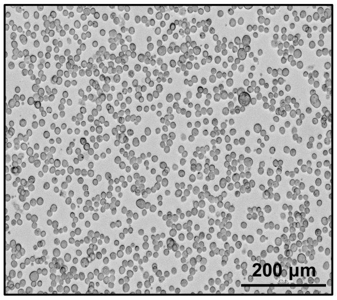

4 nM Doxorubicin

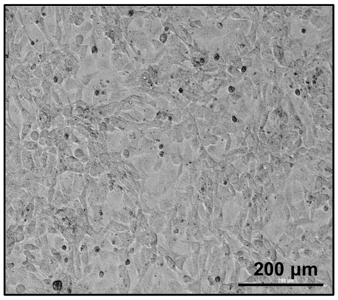

8 nM Doxorubicin

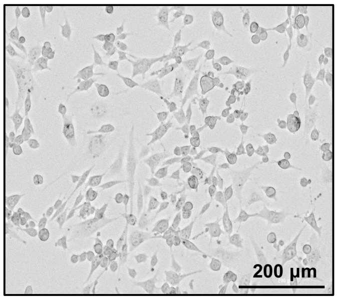

16 nM Doxorubicin

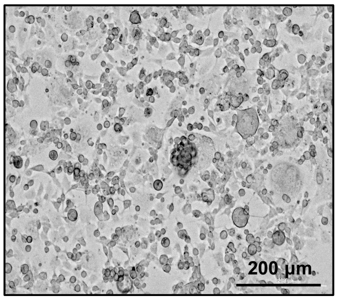

32 nM Doxorubicin

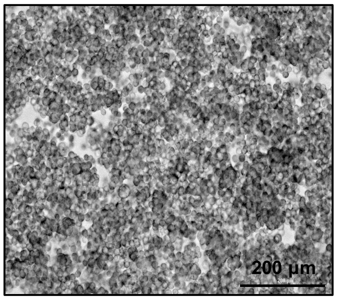

64 nM Doxorubicin

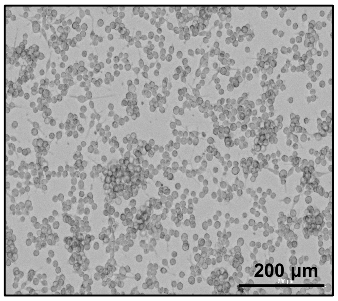

128 nM Doxorubicin

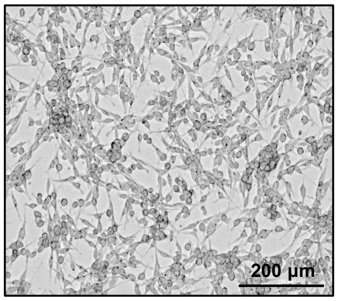

256 nM Doxorubicin

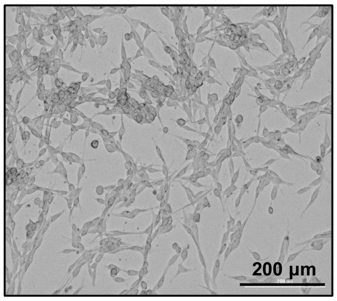

500 nM Doxorubicin

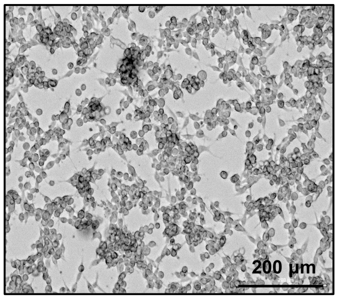

1 µM Doxorubicin

Progression of B16 MDR derived cancer cell line from B16 Parent cancer cell line

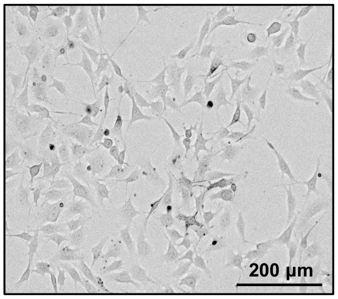

Parent B16

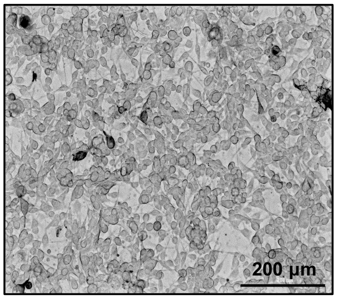

4 nM Doxorubicin

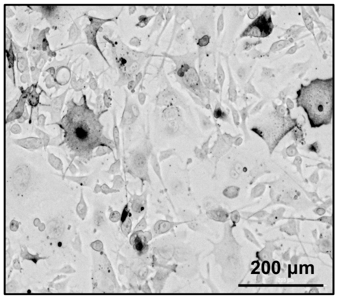

8 nM Doxorubicin

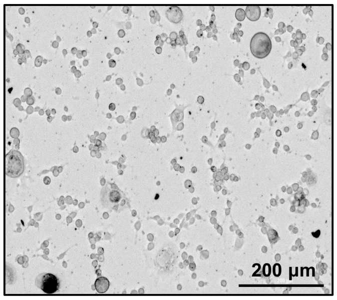

16 nM Doxorubicin

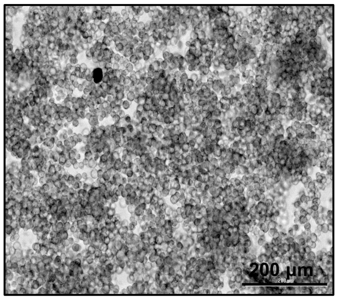

32 nM Doxorubicin

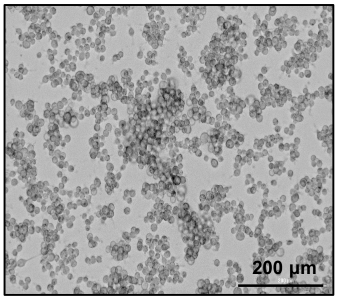

64 nM Doxorubicin

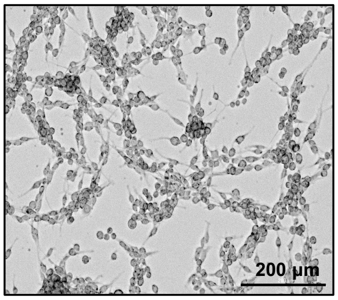

128 nM Doxorubicin

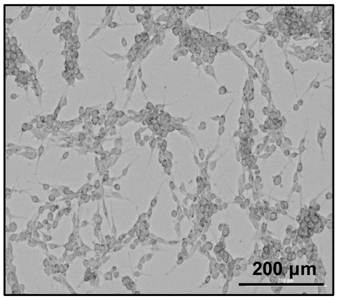

256 nM Doxorubicin

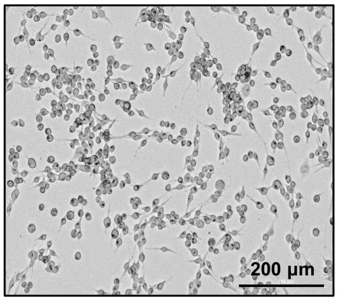

500 nM Doxorubicin

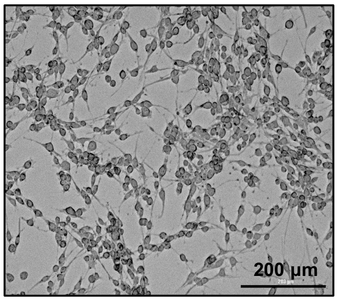

1 µM Doxorubicin

Progression of 4T1 MDR derived cancer cell line from 4T1 Parent cancer cell line

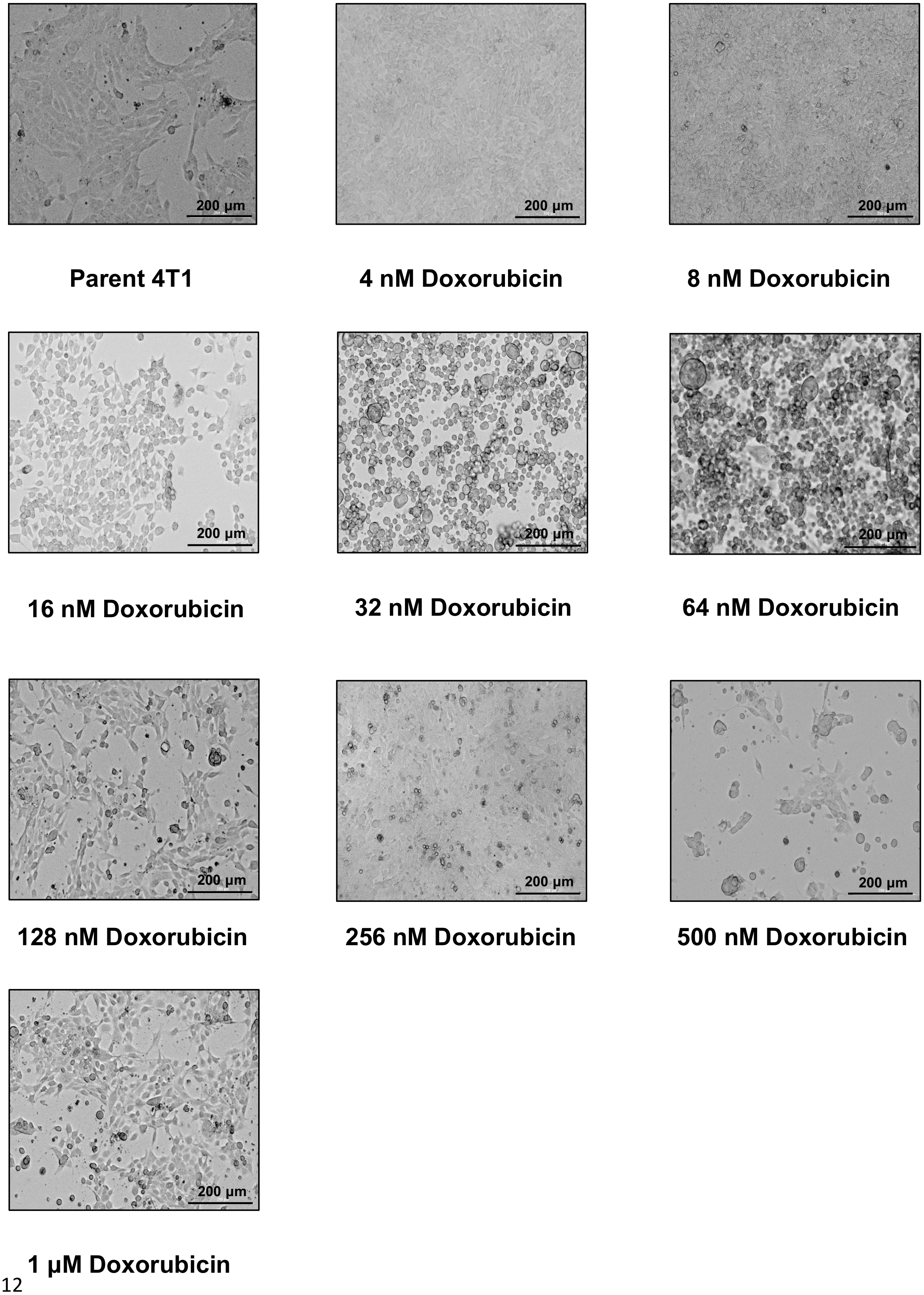

Flow cytometry histograms: competitive experiments with Rhodamine 123

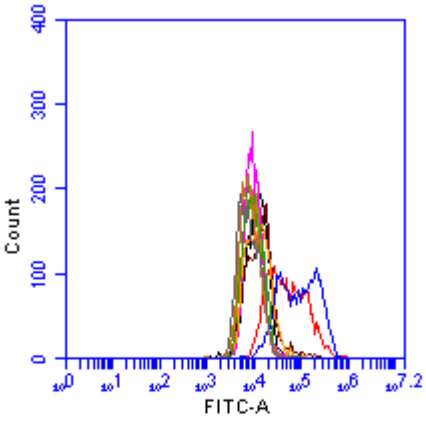

AT3B1 - MDR

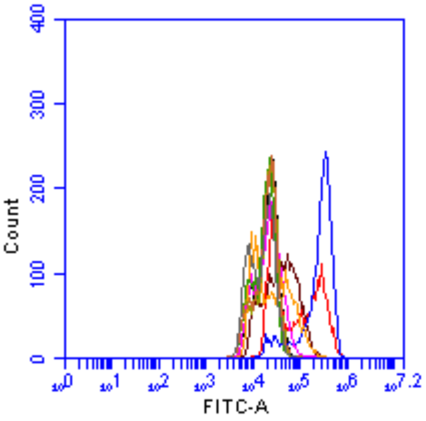

TC2 - MDR

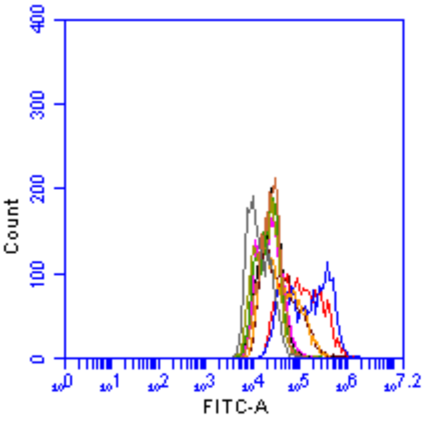

B16 - MDR

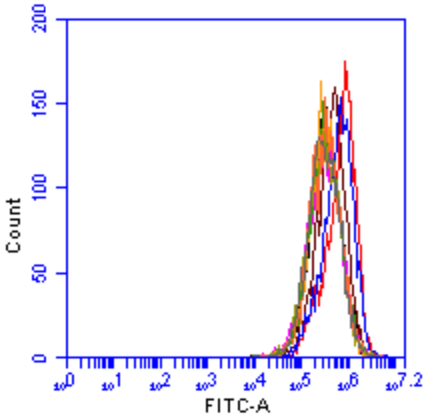

4T1 - MDR

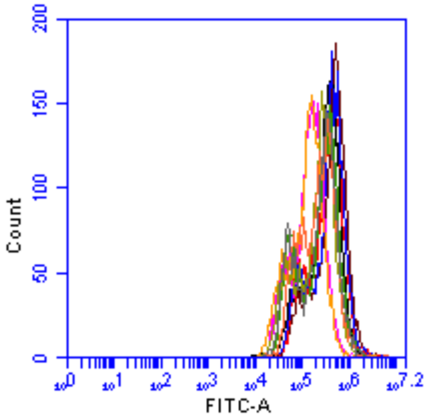

TC2 - Parent

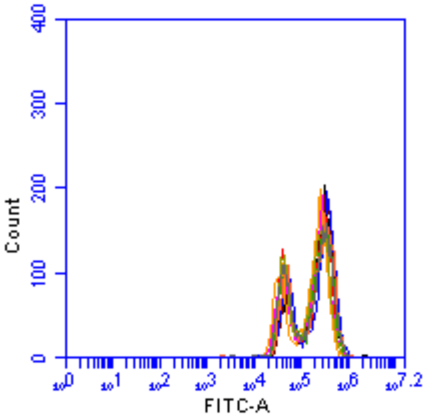

B16 - Parent

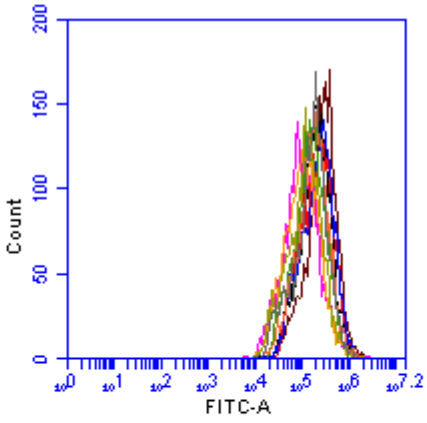

4T1 - Parent

Overlay Histogram Legend

- 1 μM Rh123
- 1 μM VER
- 10 μM VER
- 1 μM TQR
- 1 μM IMQ
- 10 μM IMQ
- 1 μM RSQ
- 10 μM RSQ
- 1 μM GDQ
- 10 μM GDQ

Flow cytometry histograms: uptake experiments with Rhodamine 123

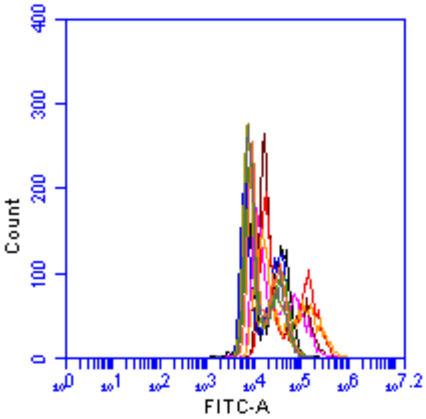

AT3B1 - MDR

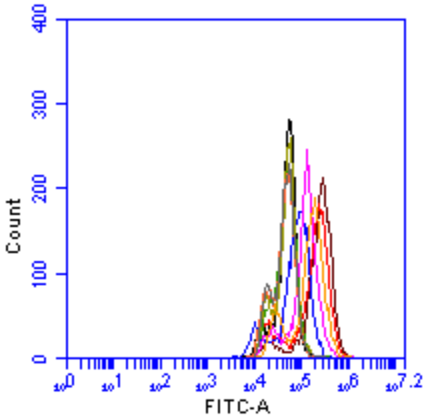

TC2 - MDR

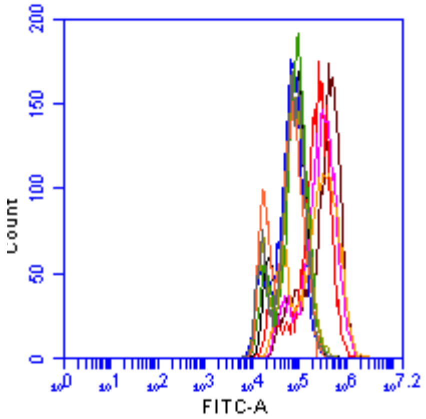

B16 - MDR

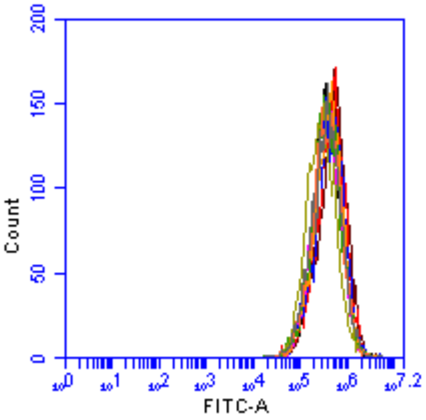

4T1 - MDR

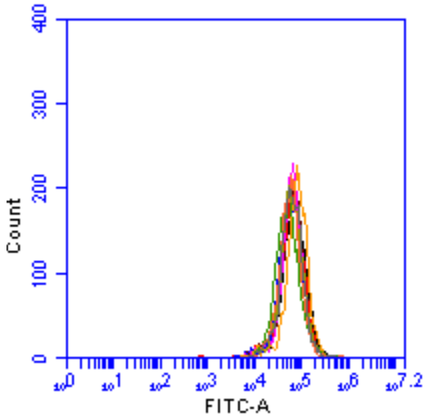

TC2 - Parent

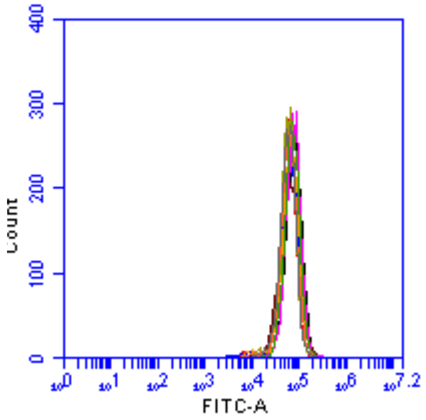

B16 - Parent

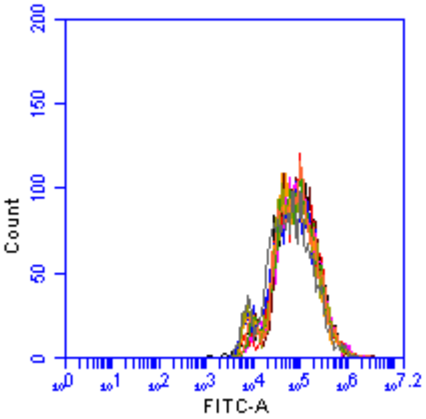

4T1 - Parent

Overlay Histogram Legend

- **1 µM Rh123**
- **1 µM VER**
- **10 µM VER**
- **1 µM TQR**
- **1 µM IMQ**
- **10 µM IMQ**
- **1 µM RSQ**
- **10 µM RSQ**
- **1 µM GDQ**
- **10 µM GDQ**

Synthetic procedure for Gardiquimod

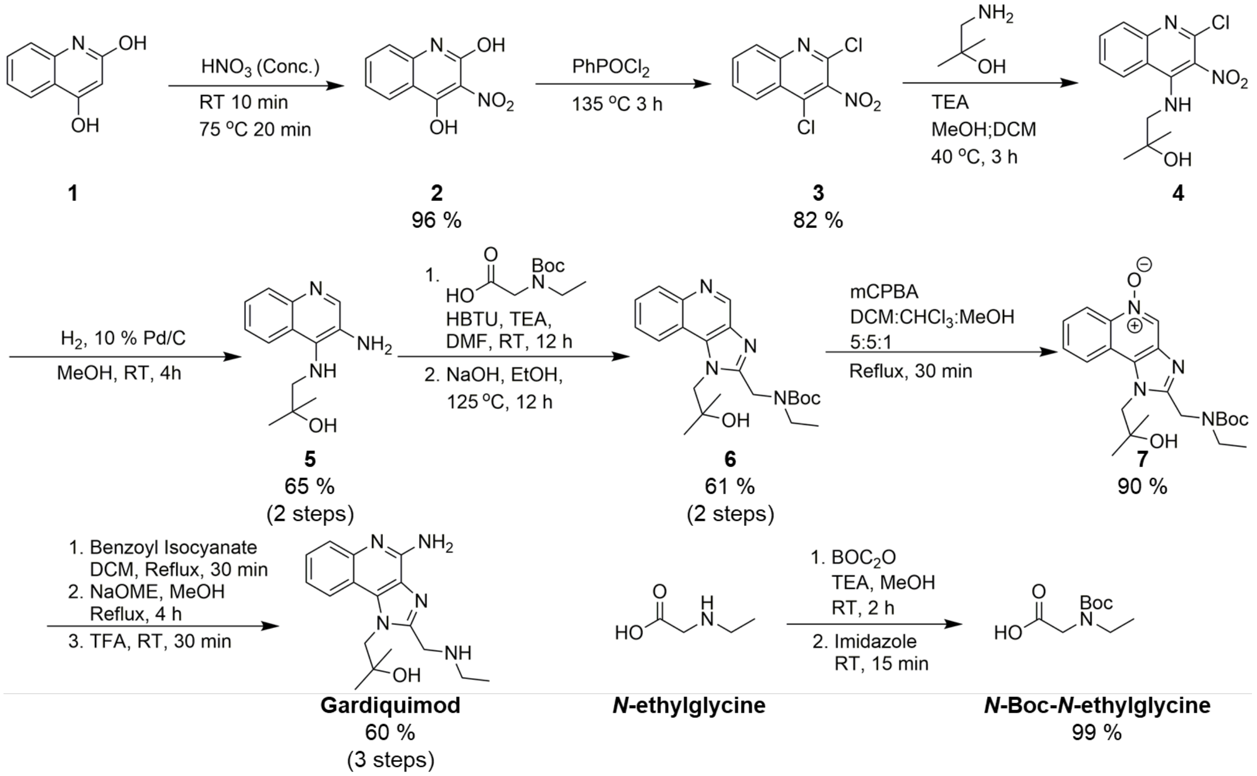

Scheme 1: Synthetic route used to prepare Gardiquimod in 17% linear yield from 2,4-dihydroxyquinoline.

The synthetic route used to synthesize Gardiquimod was adapted from the methods reported by Taguchi [1], compounds (**4** and **5**), Gerster [2] compounds (**3**), and David [3,4] compounds (**2**, **6, 7,** and Gardiquimod). 2,4-dihydroxy quinoline (**1**) was chosen as an inexpensive and readily available starting material. 2,4-dihydroxyquinoline (**1**) is nitrated in neat concentrated nitric acid (A.C.S. Grade) to yield 2,4-dihydroxy-3-nitroquinoline (**2**) which can be precipitated in ice cold H_2O. Compound (**2**) is then heated in neat phenylphosphonic dichloride at 135 ˚C to yield 2,4-dichloro-3-nitroquinoline (**3**) which can be recovered following precipitation in ice cold H_2O, trituration in benzene, and lyophilization of the organic filtrate. Compound (**3**) undergoes nucleophilic aromatic substitution under basic conditions with 1-amino-2-methylpropan-2-ol to yield the secondary amine (**4**). Although the reaction preferentially occurs at the 4 position, an amount of diamine from displacement of both chlorides also occurs, but can be separated from (**4**) by recrystallization in benzene.

At this point the nitro group is reduced by hydrogenation. The 2-chloro substituent is reduced with palladium catalyst and diamine (**5**) is obtained in good purity following removal of catalyst and evaporation of solvent. *N*-Boc-*N*-ethylglycine is activated with HBTU and reacted with diamine (**5**), after 12 h solvent is removed, and the resultant amide refluxed under basic conditions to aromatize the system to accomplish the Traube

reaction yielding imidazoquinoline (**6**) which is purified by column chromatography with isocratic Ethyl acetate with 1% TEA.

To install the aniline on the 4 position, imidazoquinoline (**6**) is oxidized with mCPBA to yield *N*^{5}-oxide (**7**). When the *N*-oxide is treated with benzoyl isocyanate in DCM at reflux the benzoyl protected aniline is provided. The benzoyl group is removed via methanolysis to liberate the anilino group and the Boc protecting group is removed with standard trifluoroacetic acid deprotection, and the resultant crude residue freebased with triethylamine and purified via a reverse phase C18 flash column using a gradient of 10 to 90 % acetonitrile in water to yield Gardiquimod in 17% linear yield over 10 steps from 2,4-dihydroxy quinoline.

Characterization of Gardiquimod

Gardiquimod

^{1}H NMR (600 MHz, DMSO-*d6*) δ 8.21 (d, *J* = 8.2 Hz, 1H; Ar-H), 7.59 (d, *J* = 8.3 Hz, 1H; Ar-H), 7.39 (t, *J* = 7.6 Hz, 1H, Ar-H), 7.21 (t, *J* = 7.5 Hz, 1H, Ar-H), 6.52 (s, 2H; NH_2), 4.70 (s, 2H; CH_2), 4.10 (s, 2H; CH_2), 2.60 (q, *J* = 7.1, 6.5 Hz, 2H; CH_2), 1.16 (s, 6H; 2 CH_3), 1.03 (t, *J* = 7.2 Hz, 3H; CH_3) ppm; ^{13}C NMR (151 MHz, DMSO-*d6*) δ = 152.8, 151.9, 145.1, 133.9, 126.3, 126.3, 126.0, 121.0, 120.5, 115.4, 70.2, 55.2, 45.6, 43.1, 27.8, 14.7 ppm; UV/VIS(Acetonitrile): $\lambda_{Max}(\varepsilon)$= 228 nm (35400), $\lambda_2(\varepsilon)$= 247 nm (34700); IR(ATR): 3301 (br, m), 2984 (m), 1673 (s), 1200 (s), 1131 (s), 721 (w); HRMS(MALDI): m/z calcd for $C_{17}H_{24}N_5O$: 314.1981 $[M+H]^+$, observed 314.19733. (Δ=2.45 ppm).

^{1}H NMR Spectra of Gardiquimod

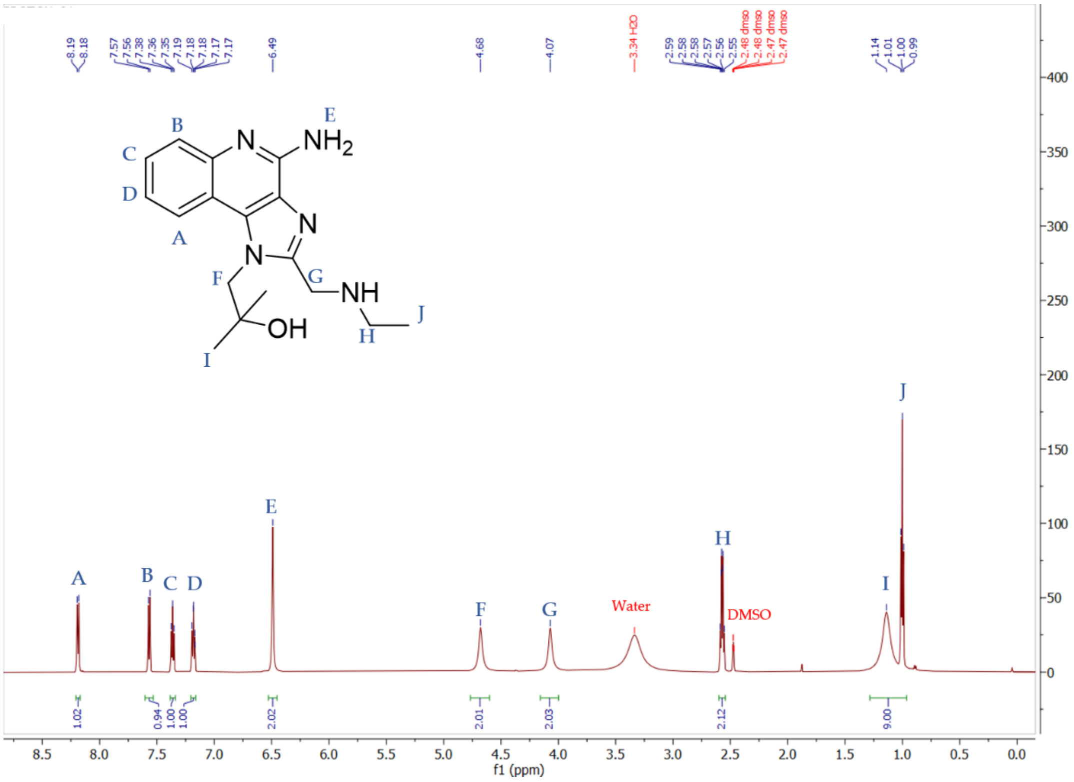

^{13}C Spectra of Gardiquimod

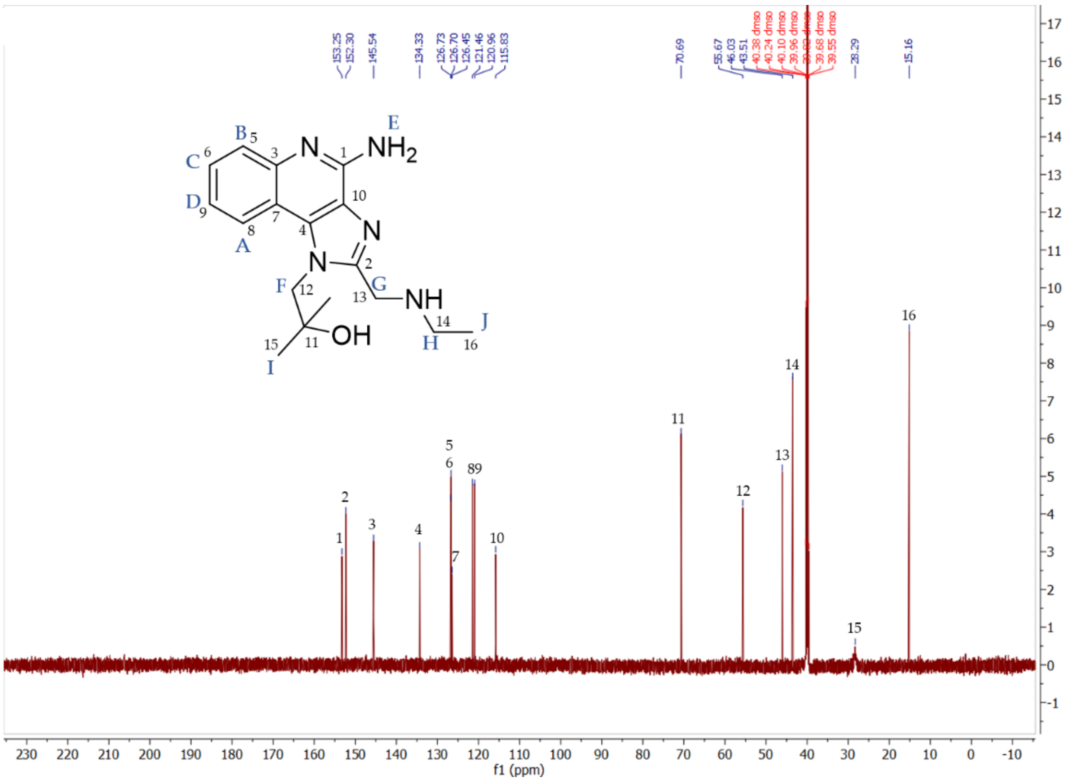

COSY Spectra of Gardiquimod

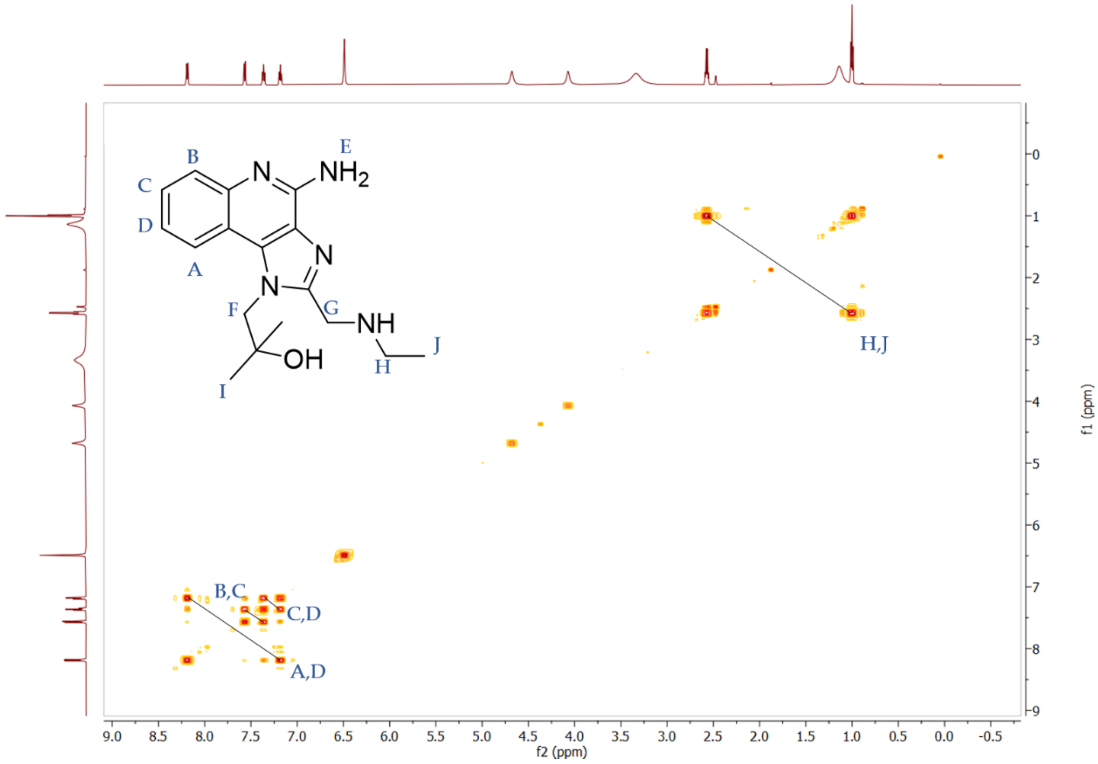

HSQC Spectra of Gardiquimod

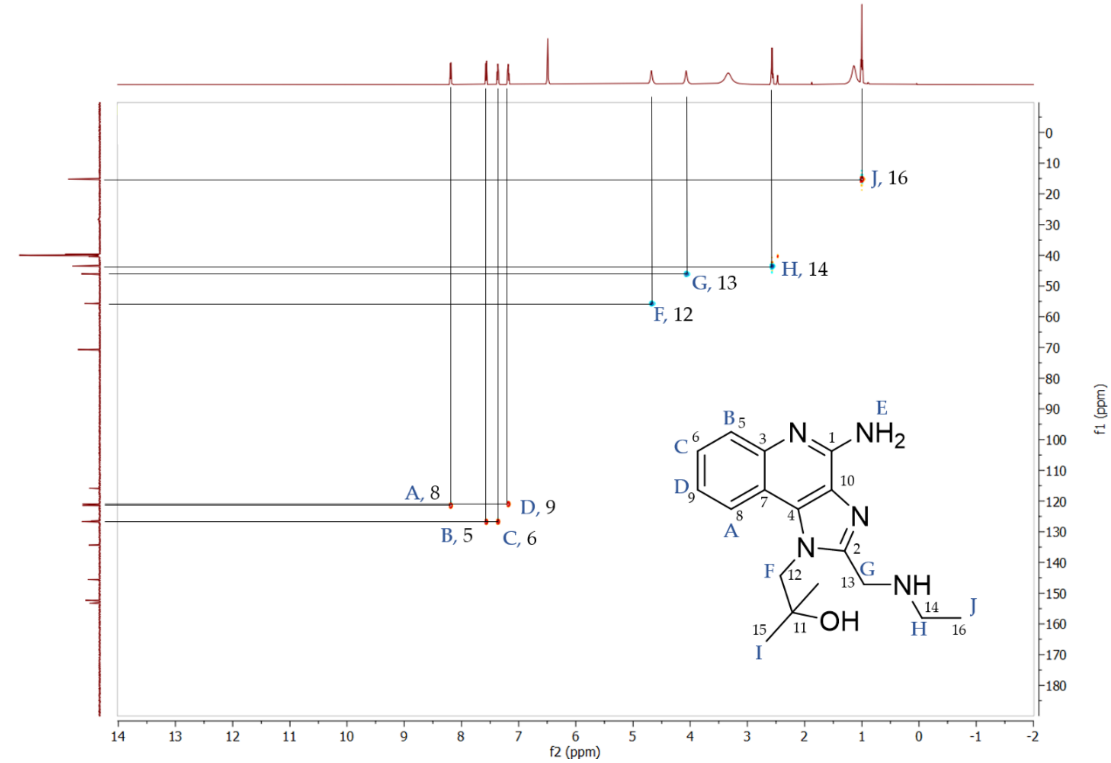

HSQC with enhanced intensity to show signal for I

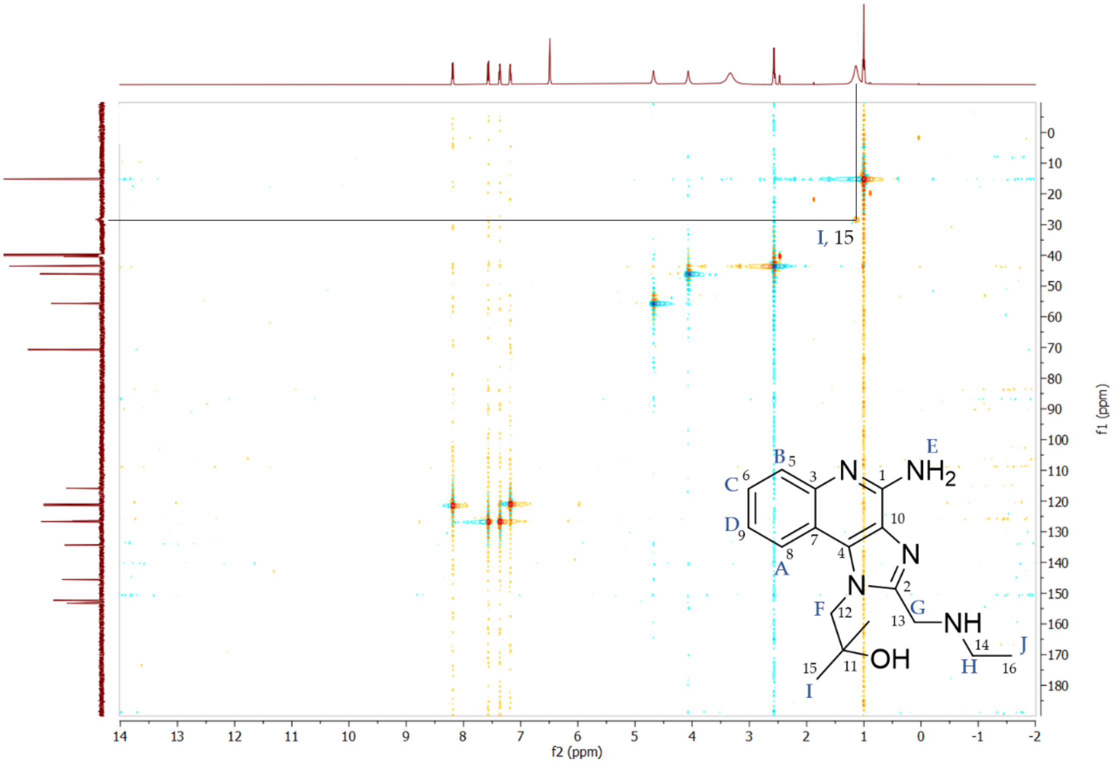

^{1}H NMR Spectra for Numbered Intermediates **2-7**

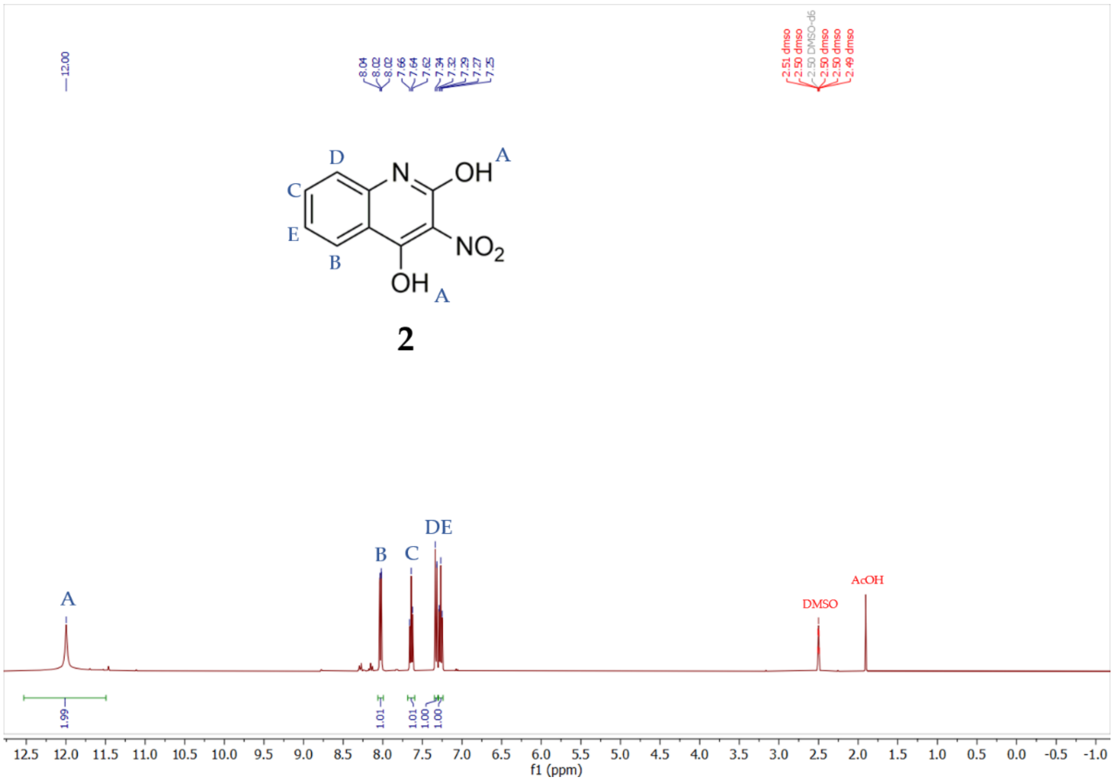

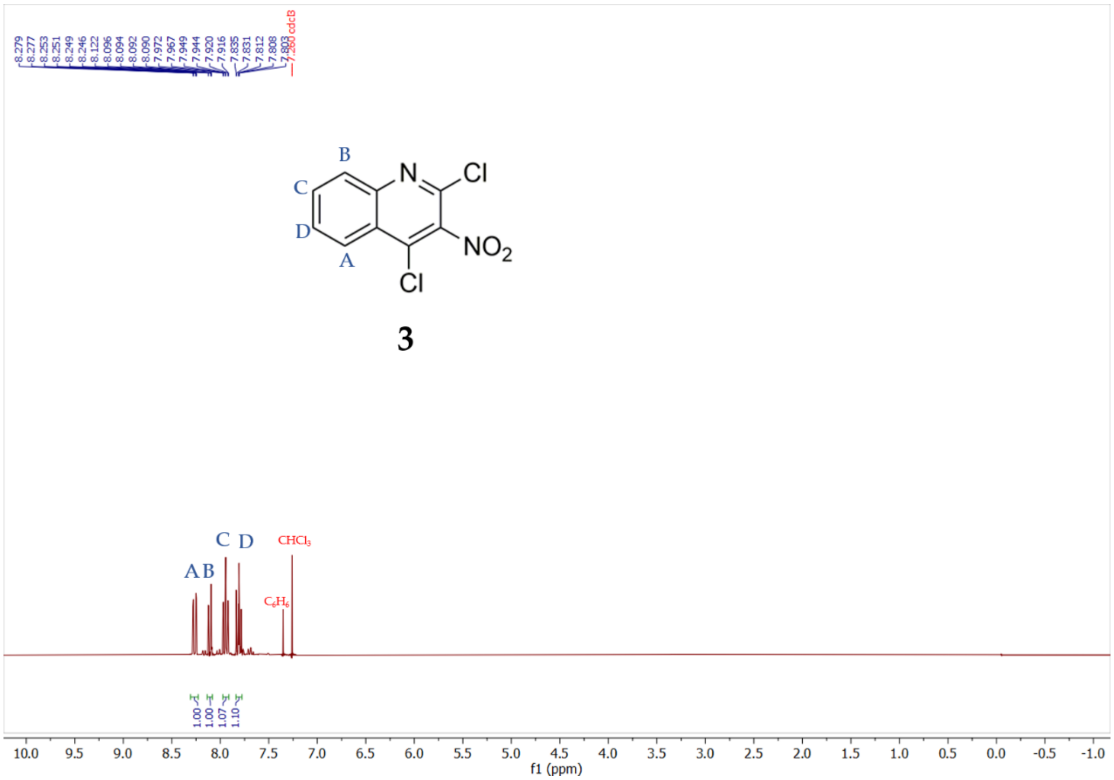

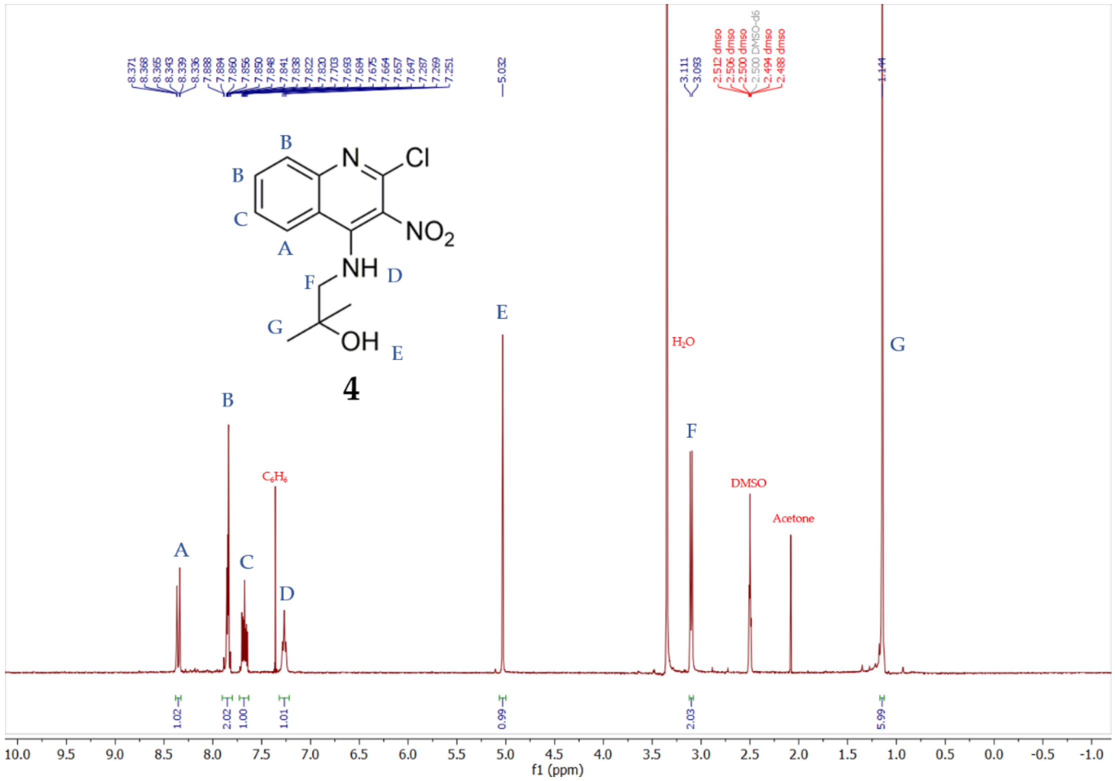
4
A
B
C
D
E
F
G
H2O
C6H6
DMSO
Acetone
f1 (ppm)

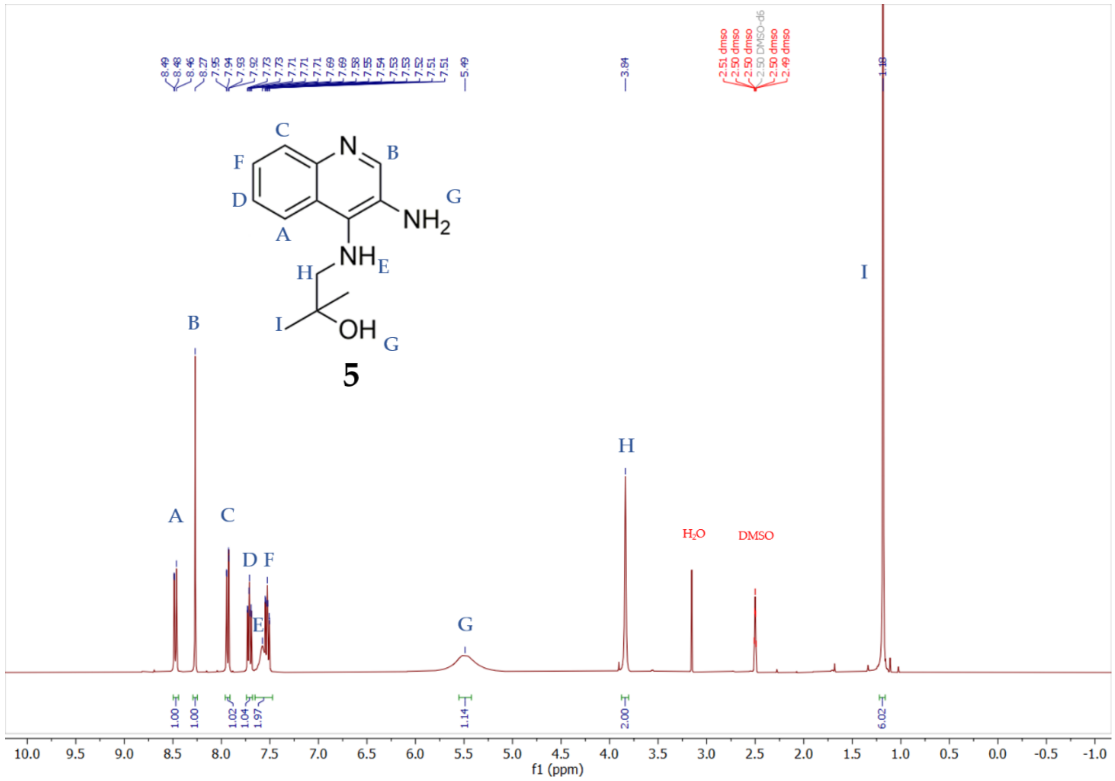
5
A
B
C
D
E
F
G
H
I
H2O
DMSO
f1 (ppm)

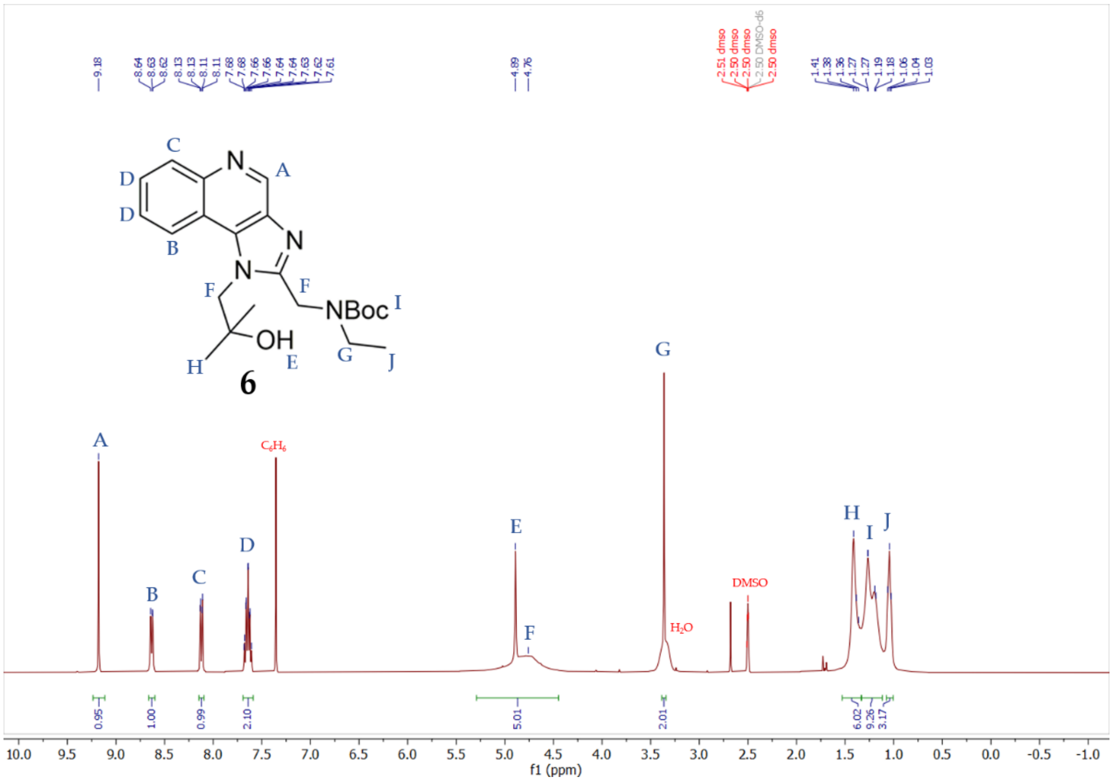
6
A
B
C
D
E
F
G
H
I
J
NBoc
OH
C_6H_6
DMSO
H_2O
f1 (ppm)

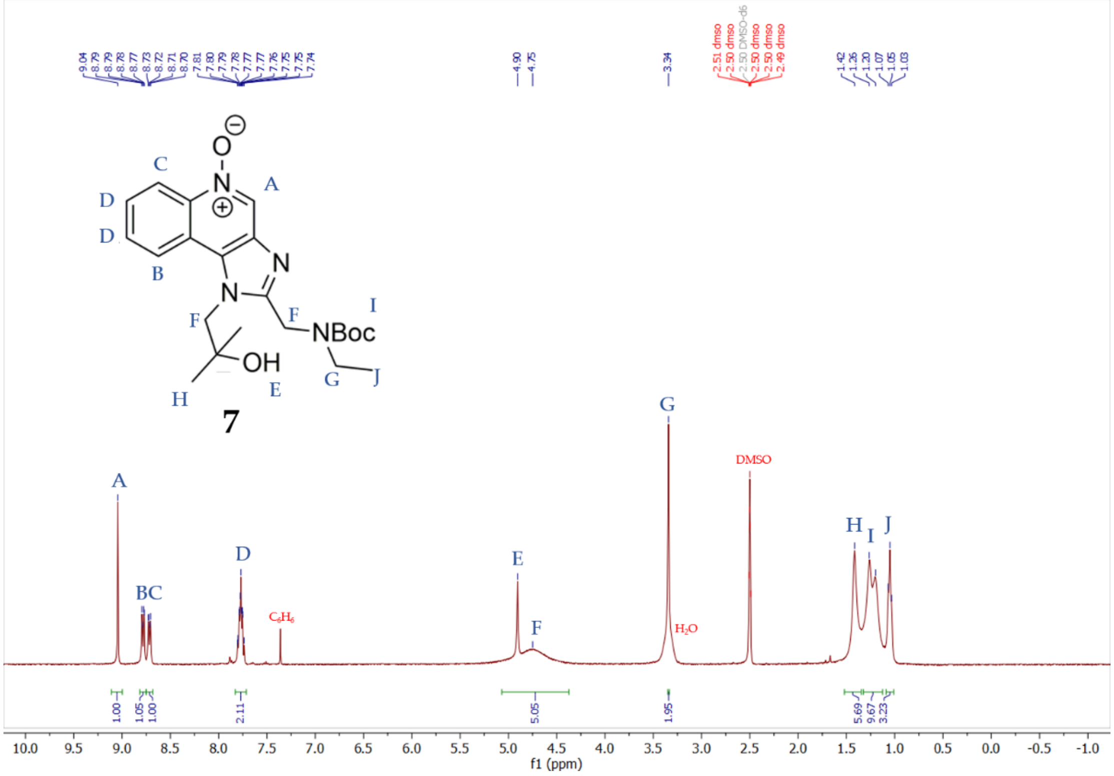
7
A
BC
D
E
F
G
H
I
J
NBoc
OH
C_6H_6
DMSO
H_2O
f1 (ppm)

References

1. Fujita, Y.; Hirai, K.; Nishida, K.; Taguchi, H. 6-(4-Amino-2-Butyl-Imidazoquinolyl)-Norleucine: Toll-like Receptor 7 and 8 Agonist Amino Acid for Self-Adjuvanting Peptide Vaccine. *Amino Acids* **2016**, *48*, 1319–1329, doi:10.1007/s00726-016-2190-7.
2. Gerster, J.F.; Lindstrom, K.J.; Miller, R.L.; Tomai, M.A.; Birmachu, W.; Bomersine, S.N.; Gibson, S.J.; Imbertson, L.M.; Jacobson, J.R.; Knafla, R.T.; et al. Synthesis and Structure−Activity-Relationships of 1H-Imidazo[4,5-c]Quinolines That Induce Interferon Production. *J. Med. Chem.* **2005**, *48*, 3481–3491, doi:10.1021/jm049211v.
3. Shukla, N.M.; Malladi, S.S.; Mutz, C.A.; Balakrishna, R.; David, S.A. Structure−Activity Relationships in Human Toll-Like Receptor 7-Active Imidazoquinoline Analogues. *J. Med. Chem.* **2010**, *53*, 4450–4465, doi:10.1021/jm100358c.
4. Shukla, N.M.; Kimbrell, M.R.; Malladi, S.S.; David, S.A. Regioisomerism-Dependent TLR7 Agonism and Antagonism in an Imidazoquinoline. *Bioorganic & Medicinal Chemistry Letters* **2009**, *19*, 2211–2214, doi:10.1016/j.bmcl.2009.02.100.
